# PhosSA: Fast and accurate phosphorylation site assignment algorithm for mass spectrometry data

**DOI:** 10.1186/1477-5956-11-S1-S14

**Published:** 2013-11-07

**Authors:** Fahad Saeed, Trairak Pisitkun, Jason D Hoffert, Sara Rashidian, Guanghui Wang, Marjan Gucek, Mark A Knepper

**Affiliations:** 1 Epithelial Systems Biology Laboratory, National Heart Lung and Blood Institute (NHLBI), National Institutes of Health (NIH), Bethesda, Maryland, USA; 2 Proteomics Core Facility, National Heart Lung and Blood Institute (NHLBI), National Institutes of Health (NIH), Bethesda, Maryland, USA; 3 Department of Electrical Engineering and Computer Science, Catholic University of American, Washington D.C, USA; 4 Faculty of Medicine, Chulalongkorn University, Bangkok, Thailand

## Abstract

Phosphorylation site assignment of high throughput tandem mass spectrometry (LC-MS/MS) data is one of the most common and critical aspects of phosphoproteomics. Correctly assigning phosphorylated residues helps us understand their biological significance. The design of common search algorithms (such as Sequest, Mascot etc.) do not incorporate site assignment; therefore additional algorithms are essential to assign phosphorylation sites for mass spectrometry data. The main contribution of this study is the design and implementation of a linear time and space dynamic programming strategy for phosphorylation site assignment referred to as PhosSA. The proposed algorithm uses summation of peak intensities associated with theoretical spectra as an objective function. Quality control of the assigned sites is achieved using a post-processing redundancy criteria that indicates the signal-to-noise ratio properties of the fragmented spectra. The quality assessment of the algorithm was determined using experimentally generated data sets using synthetic peptides for which phosphorylation sites were known. We report that PhosSA was able to achieve a high degree of accuracy and sensitivity with all the experimentally generated mass spectrometry data sets. The implemented algorithm is shown to be extremely fast and scalable with increasing number of spectra (we report up to 0.5 million spectra/hour on a moderate workstation). The algorithm is designed to accept results from both Sequest and Mascot search engines. An executable is freely available at http://helixweb.nih.gov/ESBL/PhosSA/ for academic research purposes.

## Introduction

Mass spectrometry is an essential component in modern large-scale proteomics studies for protein identification and quantification [1-3]. Mass spectrometers measure the mass-to-charge ratio (m/z) of ionized molecules [4]. Peptides are desolvated in the gas phase as ions in a typical liquid chromatography-coupled tandem mass spectrometry (LC-MS/MS) proteomics experiment. Peptide information is extracted from these ions following various fragmentation strategies such as CID (Collision Induced Dissociation) and HCD (Higher Energy Collisional Dissociation). These fragmentation methods tend to generate ostensibly similar spectra for the same peptide, but with slight differences in the presence and relative abundance of certain ions. Various search algorithm are used to match the fragmented spectra with protein databases to identify the peptides present in the sample [5,6].

Mass spectrometry based phosphoproteomics has useful biological applications such as study of cell regulation [7], cancer diagnostics & therapeutics [8] and others [1,9-13]. With the advent of high-throughput mass spectrometers that can generate large data sets, efficient and scalable analysis tools are essential for useful proteomics studies. The post-acquisition analysis of mass spectrometry data is faced with numerous computational challenges such as estimation of false discovery rate (FDR) [14], quantification of peptides [1], spectra-to-peptide matching [5,6], and identifications of post-translational modifications (PTM) [15].

Phosphorylation site assignment has been done manually in the past by looking at the spectra. However, due to large volumes of data generated from high-throughput mass spectrometers this is no longer practical. Accurate site assignment is critical for drawing biological conclusions that generally seek to identify protein kinase classes that may be regulated in an experiment as a results of phosphorylation [16]. Systematic errors in site assignment would undermine these calculations and the conclusions that arise from them. Therefore, there is a need for an efficient algorithm that can accurately assign phosphorylation sites in various kinds of mass spectrometry data sets obtained from different experimental conditions.

There have been a number of algorithms that automate the process of phosphorylation site assignment for mass spectrometry data [15,17-25]. Among them is Ascore [17], a well known algorithm that uses probabilistic analysis for phosphorylation site determination. Another commonly used algorithm is PhosphoScore [15] that uses a graph theoretic approach combined with Gibbs sampling to determine the phosphorylation sites.

Other methods include MD-score [25] which computes the ratio of the difference between the best and second best Mascot score, SLIP [23] that determines the site by comparing probability and expectation values for the same peptide with different site assignments and, SLoMo [24] modifies Ascore to make the tool capable of analyzing a wide range of spectra and peptides.

The main goal of this paper is to present a dynamic programming algorithm, PhosSA, for phosphorylation site assignment of large scale mass spectrometry data. Our objective function for the algorithm is based on the sum of peak intensities that match a particular theoretical peptide spectrum. The algorithm is designed for HCD as well as CID fragmented spectra as well as for labelled spectra such as iTRAQ and SILAC. After a score is received for each potential peptide, quality post-processing steps are invoked that exploit specific characteristics of mass spectrometry data to stratify the assigned sites based on the scores and the other associated parameters. We report high quality site assignment using PhosSA with accuracy *>*99% and high degree of sensitivity for our experimentally generated data sets with varying conditions and known phosphorylation sites. Note that Accuracy = (No. of peptides with correct assignment ÷ number of peptides that pass the post-processing criteria) *× *100 and Sensitivity = (No. of peptides that pass the post-processing criteria ÷ Total No. of peptides in the dataset) × 100. Dynamic programming allows us to design the algorithm with linear space and time complexities making it a highly useful and efficient tool for site assignments of large-scale mass spectrometry data sets. PhosSA was introduced as the first dynamic programming algorithmic solution to phosphorylation site assignment problem in [26] and this paper is an extension of that framework.

### Problem statement and background information

**Figure 1:**
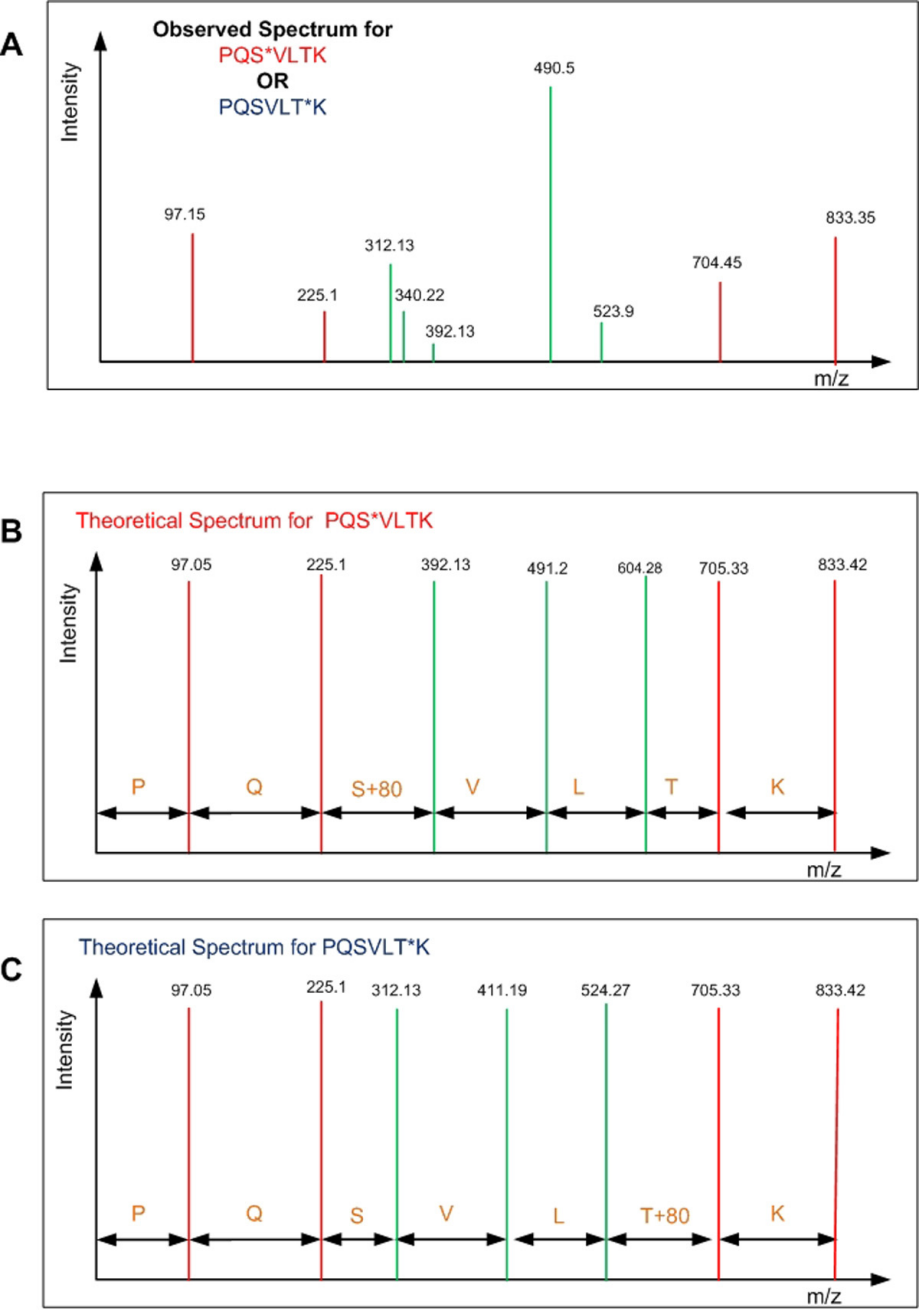
Simplified problem statement: Observed spectrum of a mono-phosphorylated peptide (PQSVLTK) is shown (A) and the problem is to determine which of the possible configuration PQS*VLTK (shown in B) or PQSVLT*K (shown in C) (* indicates phosphorylated residue) has the theoretical spectrum that corresponds best to the observed spectrum.

Let the fragmentation spectra be represented as *S *= (*m*_1_*, i*_1_), (*m*_2_*, i*_2_)*, · · · *, (*m_Q_, i_Q_*) where *m_x _*is equal to the m/z ratio of the peptide fragment and *i_x _*equals the intensity of the fragmented peak at position *x *where 0 *< × ≤ Q*, and *Q *is the total number of peptide fragments. A standard peptide search algorithm (e.g Sequest, Mascot etc.) generally perform well to match peptides from the database to the fragmented spectra. However, these algorithms have limited capability for assigning phosphorylation sites due to the similarity of the spectra from all the possible configurations of the peptide [i.e. a difference in the position of the phosphorylated residue(s)] [17,25]. There are only certain peaks, called *site determining peaks*, that can help differentiate the correct configuration among all potential configurations. The objective of the algorithm is to determine which peptide configuration has the best matching site determining peaks to the observed spectrum. Figure 1 shows an observed spectrum and two theoretical spectra of two possible phosphopeptide configurations and the problem is to determine which configuration is the correct one for the given spectra. The site determining peaks are shown as green peaks and the red peaks represent non-site determining peaks. The theoretical spectra from the two potential peptide candidates reveals the following insights: 1) Only a few peaks match the theoretically generated peaks (e.g peaks at 340.22), 2) there are more peaks in the observed spectrum as compared to the theoretical spectra, and 3) some of the observed peaks matching theoretical ones are very close to the background noise level (e.g. peak at 392.13). The differences in the fragmentation methodologies, variability in biological sample preparation, and differences in mass spectrometry instrumentation also contribute to the stochastic noise in the data, further complicating the analysis. All of these problems are frequently observed in real spectra making many post-processing tasks (e.g. database searching, site assignment etc.) difficult computational problems.

## Methods

### Algorithm: PhosSA

This section is dedicated to the details of the dynamic programming formulation of our algorithm. Mathematical formulation, quality post-processing and related time and space complexities will be discussed in this section.

**Figure 2:**
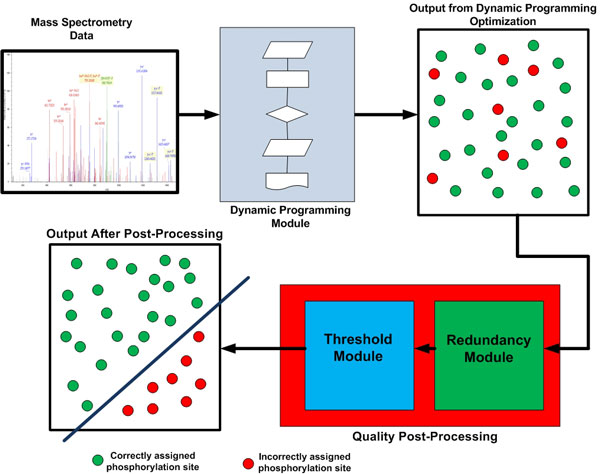
**Flow diagram for PhosSA algorithm**. Mass spectrometry data are fed into the dynamic programming module. The dynamic programming module output is the optimal assignment for each peptide, regardless of the overall quality of match. To sort the assignments by quality of match, a quality post-processing is used. The quality post-processing uses two additional criteria (Threshold (*dC_n_*) and redundancy) that allow discrimination between phosphorylation site assignment that are correctly vs. incorrectly assigned.

Sequest and Mascot search results are taken as input for PhosSA algorithm and the concept is extendible for other search algorithms such as Inspect and OMSSA [5,27]. Like any search algorithm, the search results from Sequest consists of multiple phosphopeptide configurations of each spectrum. For the sake of discussion in the paper, Sequest will be considered the primary search engine used as input to PhosSA. All the discussions and methods also apply to Mascot results. PhosSA establishes theoretical site determining peaks based on the configurations reported by Sequest and m/z ratios of those peaks are calculated. The observed spectrum is then compared to the theoretical spectra that also contains the site determining ions for each configuration. The neutral loss peaks, different charge states and random noise make the optimization task markedly complex. The intensities of observed peaks that only match theoretical site-determining peaks (within a defined m/z threshold) of each phosphopeptide configuration are summed up to a Φ score. The phosphopeptide configuration that has the highest Φ score for each spectrum is selected using dynamic programming as will be described in the manuscript. After the optimization score has been calculated and the possible configurations have been ranked in descending order of the score, a quality post-processing routine is executed. The quality post-processing is based on redundancy information in the data set and the probabilities calculated as a function of other configuration in the given spectra. An outline of the algorithm is shown in Algorithm 1 and Figure 2. The algorithm has been implemented in Java(TM) SE Runtime Environment (*build*1.6.0). The experiments were conducted on a Dell server consisting of 2 Intel Xeon(R) Processors, each running 2.40 GHz, with 12000 KB cache and 64GB DRAM memory. The operating system on the server is Linux RedHat enterprize version with kernel 2.6.9-89.ELlargesmip.

**Algorithm 1 **PhosSA

**Require: **Peptide search results from Sequest containing lists of possible phosphopeptide configurations and the corresponding spectral data (m/z and peak intensities):

**Ensure: **The correct phosphorylation site assignment:

1. Read the Sequest search results with the corresponding spectral data

2. Extract theoretical site determining peaks based on the configurations reported by Sequest and calculate m/z ratios of those peaks.

3. Compare observed spectrum to each set of theoretical site determining peak for each phosphopeptide configuration.

4. Calculate the optimal Φ score for each phosphopeptide configuration using dynamic programming.

5. Select the phosphopeptide configuration that has the highest Φ score for each spectrum as an input for the quality post-processing.

6. Classify the peptides as passed or ambiguous using the proposed quality post-processing criteria

7. Output the phosphopeptide data that exceed quality threshold based on the quality post-processing.

### Dynamic programming based phosphorylation site assignment algorithm

In this section we will mathematically formulate the site assignment problem, and present a dynamic programming strategy to determine the optimal solution.

#### Mathematical formulation

Let us consider an instance of the assignment problem for a peptide which has length *L *and has been identified from a spectrum using a search engine. Let the index of any amino acid in the peptide be represented by *i *where 1 *≤ i ≤ L *and the mass to charge ratio of an amino acid at position *i *be defined as *M *(*i*) and the mass to charge of the b-ion be represented as *M b*(*i*) up to *i*. In the same way, m/z of the y-ion is defined as *M y*(*i*) up to *i*. Now the sub-problem of calculating the theoretical m/z of the b-ion can be defined as:

Mb(i)=Mb(i-1)+M(i) (1)

Similarly, the y-ion can be defined in terms of b-ion m/z values

My(i)=Mb(L)-Mb(L-i) (2)

Now assume that the intensity of the peak that is observed for any theoretical mass M is defined as *I*[*M *] and the maximum peak that is selected for mass M is

$I{\left[M\right]}_{max}=max\left(I\left[q\right]\right)$ (3)

and the maximum peak intensity window searched is (*M − δ/*2) *≤ q ≤ *(*M *+ *δ/*2). *δ *defines the window size that is chosen based on the mass accuracy of the mass spectrometer used for the experiment.

Now let the objective function Φ(*L*) that has to be optimized (maximized) for a single peptide of length *L *be

$\Phi \left(L\right)=\overset{L}{\underset{u=1}{\sum }}I{\left[Mb\left(u\right)\right]}_{max}+\overset{L}{\underset{r=1}{\sum }}I{\left[My\left(r\right)\right]}_{max}$ (4)

The rationale for the objective function is that the intensity of the peaks provides weighting for the peaks that match the theoretical spectra; since the peaks with larger intensities represent peaks that are real peaks and not just random noise. The same objective function is defined for other fragmentation ions such as neutral losses of phosphoric acid (P), *H*_2_*O *and *N H*_3_. Now let a set *S *be defined as *S *= {*b*, *b − H*_2_*O*, *b − NH*_3_, *b − P *, *b − P − H*_2_*O*, *b − P − NH*_3_, *y*, *y − H*_2_*O*, *y − NH*_3_, *y − P *, *y − P − H*_2_*O*, *y − P − NH*_3_}; where S corresponds to all possible fragmentation ions. Here the notation *b − × *(x corresponds to *H*_2_*O, NH*_3 _and phosphoric acid(P)) is used to indicate the m/z of a b-ion minus the m/z corresponding to neutral loss of *H*_2_*O *(18/z), *NH*_3 _(17/z), or phosphoric acid (98/z). We define y-ions in a similar fashion. The fact that we have to identify and match neutral loss peaks, increases the search space many fold and substantially complicates the optimization problem. The effect of including water, ammonia and phosphoric acid loss on site assignment quality for CID and HCD data sets is shown in Additional File 1 (Figure S1) and Additional File 2 (Figure S2), respectively. Preliminary analysis of our data indicated that the fragment ions with neutral loss of phosphoric acid were crucial to the site assignment of HCD data (Additional File 2). Since neutral loss peaks have useful information that can be used to assign sites, they must be incorporated in algorithmic design. However, a naive implementation of the defined score (Equation 4) for each peptide under consideration will makes the time complexity equal to *O*(*L*^|*S*| × *charge*^) for each peptide, where *charge *is the charge state on the peptide and *|S| *is the number of elements in the set. For the set *S *defined earlier, the time complexity then approaches *O*(*L*^12 × *charge*^); which for a multiple charge state peptide would make the running time intracTable For example, for a +3 charge state of the peptide, the number of ions would approach *O*(*L*^36^) for a single peptide and for N peptides the running time would be as asymptotic to *O*(*N L*^|S| × charge^) ≈ *O*(*NL*^36^). Clearly, this is not feasible even for small number of peptides. However, we will show how the problem can be solved more efficiently in linear-time using our dynamic programming formulations using sub-problems from the fragmentation ions (Set S). This makes PhosSA well suited to be used with large mass spectrometry data sets.

*Dynamic programming *: We apply the standard dynamic programming approach as formulated for many problems in [28]. In order to find the optimal solution for the peptide of length *L *for {1, 2*, · · ·, L*} one has to look at the optimal solutions of sub-problems of the form {1, 2*, · · ·, j*} where *j < L*. Thus, for any value *j *from 1 and *L − *1, let *O*_(*j*) _denote the optimal solution of the problem for peptides size from {1*, · · ·, j*} and let Φ(*j*) denote the value of this solution. The optimal solution we are trying to establish is Φ(*L*). For optimal solution *O*_(*j*) _on {1, 2*, · · ·, j*} either *j ∈ O*_(*j*) _(*j *belongs to the optimal solution) in which case

$\text{Φ}\left(j\right)=I{\left[M\left[j\right]\right]}_{max}+\text{Φ}\left(j-1\right)$ (5)

or *j *∉ *O*_(*j*) _(*j *is not part of the optimal solution) in which case *O*_(*j*) _= *O*_(*j−*1) _and therefore Φ(*j*) = Φ(*j − *1). Since, there are only two possibilities, we can further say:

$\text{Φ}\left(j\right)=\text{max}\left(I{\left[M\left[j\right]\right]}_{max}+\text{Φ}\left(j-1\right),\text{Φ}\left(j-1\right)\right)$ (6)

The formulated solution *O*_(*j*) _is part of the optimal solution if and only if:

$I{\left[M\left[j\right]\right]}_{max}+\text{Φ}\left(j-1\right)\geq \text{Φ}\left(j-1\right)$ (7)

and

$I{\left[M\left[j\right]\right]}_{max}\geq \zeta$ (8)

**Figure 3:**
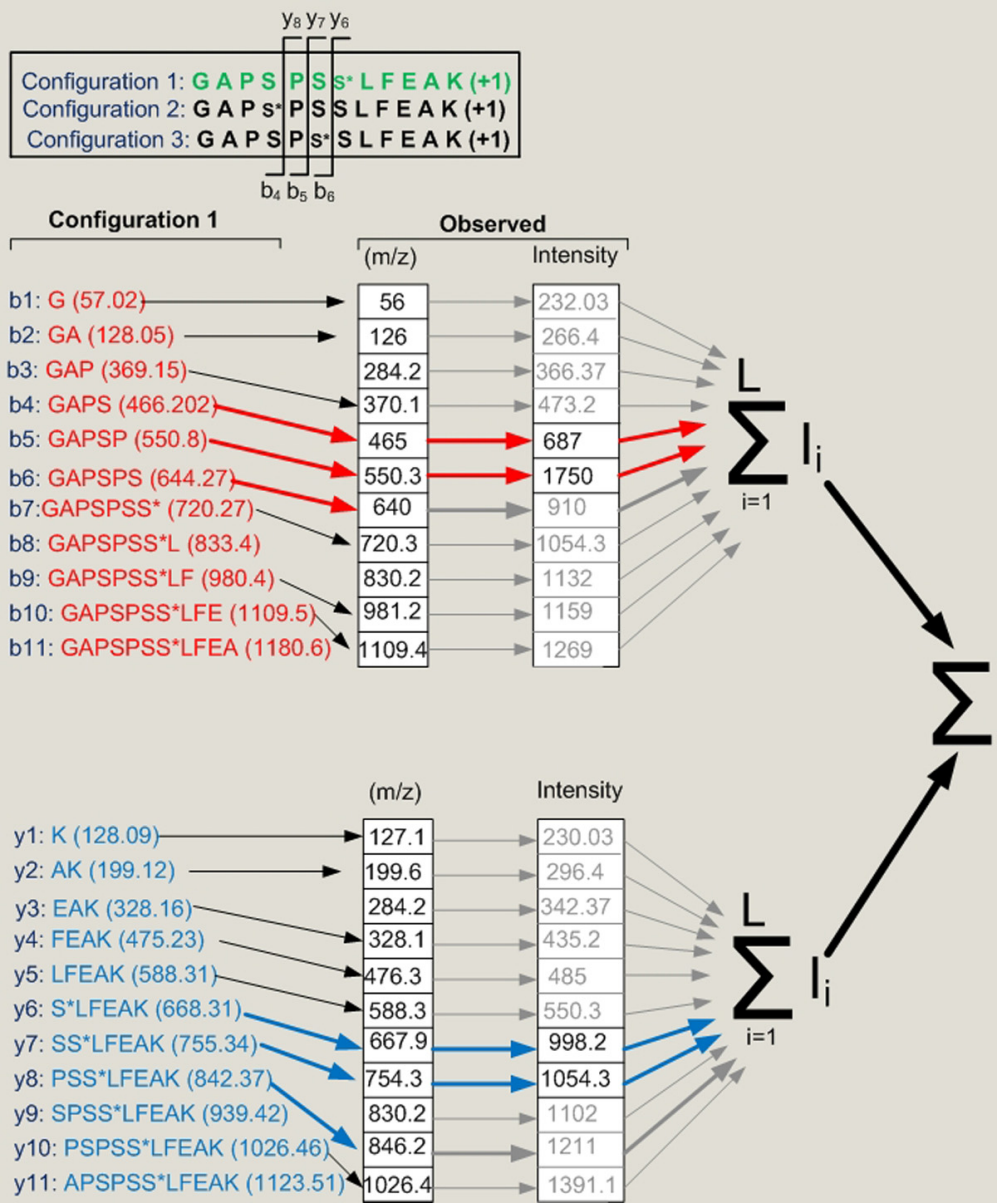
**A diagram showing the concept behind the dynamic programming algorithm**. Out of three candidate configurations, the site determining ions i.e. those fragment ions that would be specific for a particular phosphopeptide configuration, were established (*b*_4 _to *b*_6 _and *y*_6 _to *y*_8_, shown in top panel). The theoretical m/z of configuration 1 are shown. The observed m/z that only match (within a specified mass tolerance) to the theoretical site determining ions are then selected (indicated by red and blue arrows). The intensities of these site determining ions are then summed for each phosphopeptide configuration considered. Note that this diagram oversimplifies the problem because it ignores multiple charge states and neutral losses as discussed in text.

*ζ *is a threshold defined for the smallest peak intensity considered and depends on the fragmentation spectra being considered. The initial conditions for the iteration are Φ(0) = 0 and *I*[0] = 0. The peaks should be at least 3% and 5% of the maximum height peak in the spectra for HCD and CID respectively. One instance of the algorithm is depicted in Figure 3.

*Additional algorithmic constraints: *Apart from the formulation of the problem described above, there are additional constraints that have to be in place for the algorithm. These additional constraints are in place because of the possibility of two or more fragmentation ions having the same m/z values. Additional File 3 (Figure S3) shows a *M S*^2 ^spectrum with the peaks matched to two or more theoretical fragment ions highlighted by red circles.

For the sake of discussion we consider the coincidence of two fragmentation ions that have the same m/z ratio and the same argument can be extended to more than two ions. The constrains that are added are as follows:

1. If neither of the peaks is site determining, do not consider these ions further in the algorithm.

2. If one of the ion is site determining and other is not, discard both of the ions. The reason for discarding is that if only one of the ions that have the same m/z is site determining and the other is not, there is no way to know which ion has contributed more towards the peak intensity.

3. If both of the peaks are site determining, consider the peak intensity made up of both of the ions since they are predicting (or weighing) for the same phosphorylation site.

*Window Size: *The design of the algorithm dictates that the size of the peak selection window would effect the accuracy i.e. if incorrect peaks are selected during dynamic programming it would affect the accuracy of the assignment. Modern mass spectrometers are precise and accurate and the observed peaks generally do not shift more than the mass accuracy of the instrument (e.g. 0.05 Da for an Orbitrap mass spectrometer). Therefore, we have chosen the window size to be equal to the mass accuracy of the instrument for PhosSA. In order to test the effect of the window size on the accuracy and sensitivity of PhosSA, we ran the algorithm with different window sizes on the phosphopeptide library discussed in the text. The results are shown in Additional File 4 (Figure S4). As can be seen in the figure, the accuracy is the highest when the window size is equal to the mass accuracy of the machine. The accuracy drops when the window size is too stringent (genuine peaks do not get included) or too wide (additional peaks get included). The effect on sensitivity is negligible when window sizes are too stringent. Therefore, the window size is set to be the same as the mass accuracy of the instrument in our implementation.

#### Quality post-processing

After the dynamic programming stage, each peptide is scored. These scores represent varying levels of confidence in the assignment of the sites. In order to automate the process of selecting the peptides that have highest confidence assignment we have formulated post-processing steps to filter the data and tag the lower confidence assignments. To accomplish these goals we incorporated two steps, namely threshold and redundancy analysis.

*Threshold criterion: *The threshold criteria that we use is similar to the *deltaCn *(*dCn*) metric used by Sequest and other algorithms [5,6,29,30]. We defined the *dC_n _*threshold as follows:

$dC_{n}=\frac{\text{Φ}{\left(L\right)}_{Highest}-\text{Φ}{\left(L\right)}_{SecondHighest}}{\text{Φ}{\left(L\right)}_{Highest}}$ (9)

The higher the *dC_n _*value the more confident one can be in the site assignment. Two peptides getting closer Φ score will result in lower *dC_n _*value. By definition, lower *dC_n _*suggests that the scores of the two top candidates are close to one another and it is difficult to decide if the higher scoring peptides assignment is correct. However, based on a preliminary test on a set of known phosphopeptides (see Additional File 5 (Figure S5)), only 22% of the data contained high enough *dC_n _*values (*>*0.9) to confidently assign the correct site with 95% accuracy.

Additional File 6 (Figure S6) shows results from multiple spectra of a peptide with a known phosphorylation site correctly assigned using our dynamic programming algorithm. Using just the threshold (*dC_n_*) criterion it can be seen that the spectra in red would be eliminated just because the second best score was very close to the best score. We devised an additional criterion that would take into account situations like this in which we have a high degree of redundancy.

*Redundancy criterion: *It is common for abundant peptides in a mass spectrometry experiment to get selected multiple times for fragmentation [31-33]. Therefore, there is additional information that can be exploited for our post-processing strategy i.e. if the same peptide with same phosphorylation site is identified multiple times using our score, it has a higher probability of being correct irrespective of the *dC_n _*of the spectra.

PhosSA exploits this information to decide the confidence of the assignment. A simple analysis would reveal that if the same peptide from different scan numbers has scored highest, with the same site more than 7 times, then the probability that it is correct is greater than 99%. In order to establish this experimentally, we analyzed the assignment accuracy of a phosphopeptide library using *only *the redundancy criteria (Additional File 7 (Figure S7)). As can be seen from the graph that the accuracy of the assignment increases in accordance with the increasing redundancy metric and is around 100% when the metric is 4 or more. Although empirical evidence suggests that the accuracy of 100% is reached before the redundancy of 7 or more is reached, we conservatively set the redundancy at 7 or more. The final post-processing criteria for PhosSA is set as follows: if a phosphopeptide configuration has a redundancy of 7 or more, we let this configuration pass without considering *dC_n_*. If the redundancy metric is less than 7, a phosphopeptide needs to have *dC_n _*more than 0.99 (or user defined *dC_n_*) in order to pass the quality post-processing.

### Analysis of computational and memory complexity

The time complexity of PhosSA can be defined as a combination of two steps. The first part is the dynamic programming part of the algorithm which will run in *O*(*L*) times, where *L *represents the average length of the peptides; making the total time complexity *O*(*N L*), since there are *N *peptides in total. The second part of the complexity is dictated by the post-processing criteria that PhosSA uses to assign confidence in the site assignment. This post-processing procedure has two parts as well: the first part is the time to calculate *dCn *which can be computed in constant time *O*(*c*). The second part of the post-processing criteria is calculation of redundancy in the data; this can be run in *O*(*kN *) time where *k *is the number of times a peptide appears with same phosphorylation site. Summing these individual complexities will reveal the linear time complexity of PhosSA algorithm equal to *T *(.) = *O*(*N L *+ *c *+ *kN *) *≈ O*((*k *+ *L*)*N *). The memory complexity is lower bounded by the redundancy module which requires all peptides to be in the memory, making the total memory complexity equal to *O*(*N L*).

### Sample preparation and mass spectrometry analysis

In order to assess the quality of site assignment that can be accomplished using PhosSA we used synthesized phosphopeptides with known phosphorylation sites. The algorithm was tested on data obtained by mass spectrometry with a number of variables: 1) different fragmentation methodologies (HCD vs CID), 2) varying peptide amounts, 3) total number of phosphorylation sites, and 4) the position of the potential phosphorylation sites within the peptide. These experiments allowed us to simulate a variety of experimental conditions encountered in "real world" samples.

The experiments to produce the data for PhosSA quality assessment were done in accordance with animal protocol by the Animal Care and Use Committee of National Heart Lung and Blood Institute (NHLBI), NIH ACUC protocol No. H-0110. A piece of freshly isolated rat liver was minced and sonicated in guanidine-HC1(6M,3ml). The samples were then spun at 16000 *× g *to pellet the cellular debris and cleared liver lysate was reduced and alkylated [1]. A peptide standard corresponding to the C-terminal sequence of the water channel Aquaporin-2 (AQP2) from rat, (Biotin-LC-CEPDTDWEEREVRRRQS*VELHS*PQSLPRGSKA) phosphorylated at both *S*256 and *S*261 was added to 500 µg aliquots of liver sample (prior to trypsinization) with distinct amounts of 0.2 nmol, 20 pmol and 2 pmol and was named AQP2-H-(S256/S261), AQP2-M-(S256/S261), AQP2-L-(S256/S261) respectively. The same procedure as above was repeated for another AQP2 peptide standard (Biotin-LC-CEPDTDWEEREVRRRQSVELHSPQS*LPRGSKA) phosphorylated at *S*264, with amounts of 0.2 nmol, 20 pmol and 2 pmol and was named AQP2-H-(S264), AQP2-M-(S264), AQP2-L-(S264) respectively. Peptide samples were desalted on a 1 ml HLB cartridge and phosphopeptides were enriched via IMAC, Pierce Phosphopeptide Isolation Kit. Samples were then desalted using *C*_18 _Ziptips (Millipore) and then were dissolved in 0.1% formic acid prior to analysis by mass spectrometry.

We analyzed the data using an Agilent 1100 nanoflow system LC (Agilent Technologies) connected to an Orbitrap LTQ Velos mass spectrometer (Thermo Scientific, San Jose, CA). The samples were run using optimized parameters for CID at 35% and HCD at 45% normalized collision energy. A detailed analysis of collision energy (CE) optimization for HCD fragmentation for phosphopeptides is shown in Additional File 8 (Figure S8). MS spectra were analyzed using Proteome Discoverer version 1.2 software running the Sequest search algorithm. Spectra were searched against a Rat RefSeq database with the following parameters for CID as well as HCD samples: Max. missed cleavage = 3, precursor mass tolerance = 25 ppm, fragment mass tolerance = 0.05 Da, static modification: carbamidomethyl (C: +57.021 Da), dynamic modifications: Phospho (S,T,Y: +79.966), Deamidation (N,Q: +0.984), Oxidation (M: +15.995). Each of the samples were run separately with CID as well as HCD fragmentation. The same parameters were used for Mascot searches.

## Results

We divided the performance evaluation of PhosSA into four distinct parts. First three parts assess the accuracy and sensitivity of the algorithm for phosphoproteomics data sets with known phosphorylation sites. This quality assessment allowed us to determine the performance of PhosSA with varying fragmentation methods (CID vs HCD), with varying molarities of the sample, with varying difficulty level of site assignments, with varying search engines (Sequest and Mascot), and comparison of PhosSA to other assignment tools (Ascore and PhosphoScore). The last part of the assessment section deals with traditional performance metrics such as execution time and memory requirements.

### Assessing the sensitivity and accuracy using PhosSA for HCD and CID data sets and the effect of different amounts of phosphopeptide standards

We generated data using LC-MS/MS for 6 samples (see methods section) and each sample was run using HCD as well as CID fragmentation strategy (results from PhosSA shown in Table 1). Both CID and HCD generate b- and y-type ions but there are substantial difference in the relative abundance of multiple type of netural loss ions. We used both CID and HCD fragmentation methods to assess the accuracy and sensitivity of our site assignment algorithm. The effect of using different fragmentation methods on site assignments has been shown recently in some studies [34].

**Table 1:** Summary of PhosSA, PhosphoRS and Sequest site assignment results for AQP2 mass spectrometry data set using CID and HCD fragmentations (see text for description). DNP denotes did not pass post-processing criteria

CID data sets	PhosSA sensitivity(%)	PhosSA accuracy (%)	Sequest accuracy (%)	PhosphoRS accuracy(%)
AQP2-H-(S256/S261)	91.6	100	94.6	66.7

AQP2-M-(S256/S261)	90.7	100	94.4	66.7

AQP2-L-(S256/S261)	0	DNP	100	100

AQP2-H-(S264)	91.5	100	100	100

AQP2-M-(S264)	50	100	88.8	60

AQP2-L-(S264)	0	DNP	60	50

HCD data sets				

AQP2-H-(S256/S261)	92.2	100	94.5	66.7

AQP2-M-(S256/S261)	93.3	100	96	66.7

AQP2-L-(S256/S261)	64.3	100	100	100

AQP2-H-(S264)	97.7	100	74.2	75

AQP2-M-(S264)	93.1	100	72.4	75

AQP2-L-(S264)	0	DNP	50	50

These data sets were run using PhosSA with quality threshold (*dC_n_*=0.99) and the results are shown in Table 1. As shown for CID, PhosSA has greatest sensitivity with higher peptide molar amounts, and for HCD the sensitivity is higher for all molar amounts when compared to CID samples. Low molar amounts of peptides results in sensitivity drop both for CID and HCD data sets due to the low S/N ratio spectra. The accuracy of PhosSA for all data sets was 100%. The accuracy of the site assignment for the top candidate peptide using Sequest is lower when compared with PhosSA as shown in Table 1. PhosSA results are also compared to PhosphoRS [22], a site assignment algorithm that comes pre-packaged with Proteome Discoverer Software. The site assignment results using PhosphoRS with CID and HCD data sets are also shown in Table 1.

**Figure 4:**
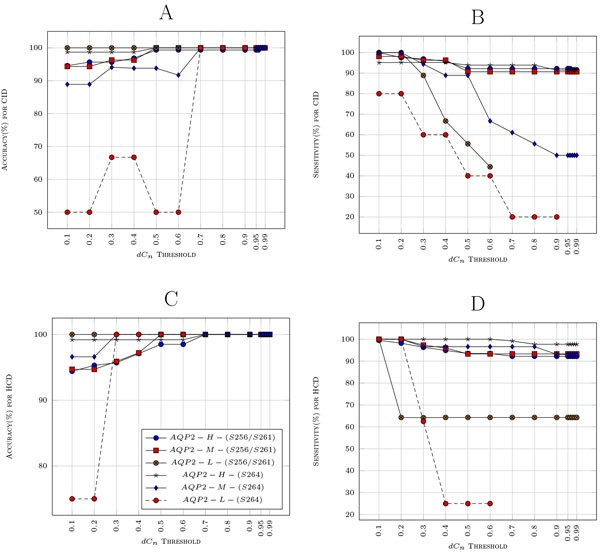
**Sensitivity and Accuracy for the 6 samples are shown with varying (dCn) threshold: A shows the accuracy and B shows the sensitivity for CID samples**. AQP2-H-(S256/S261), AQP2-M-(S256/S261) and AQP2-L-(S256/S261) consists of phosphopeptide standard (Biotin-LC-CEPDTDWEEREVRRRQS*VELHS*PQSLPRGSKA) phosphorylated at both *S*256 and *S*261 in distinct amounts of 0.2 nmol, 20 pmol and 2 pmol respectively. AQP2-H-(S264), AQP2-M-(S264) and AQP2-L-(S264) consists of peptide standard (Biotin-LC-CEPDTDWEEREVRRRQSVELHSPQS*LPRGSKA) phosphorylated at *S*264 in amounts of 0.2 nmol, 20 pmol and 2 pmol respectively.

PhosSA algorithm's redundancy threshold remains constant at 7 but allows users to set the *dCn *threshold. By definition, the lower the *dC_n _*number the less confident PhosSA is about its assignment i.e. if the user sets the *dC_n _*too low, a larger number of peptides would pass at the expense of accuracy. The optimal *dC_n _*that maximizes the number of peptides that get past the filter while maximizing the accuracy is dependent on the data set. We performed experiments while varying the *dC_n _*threshold from 0.10 to 0.99 and report both the accuracy and sensitivity shown in Figure 4. As can be seen with *dC_n _*threshold values (0.70 *− *0.99), the accuracy is almost 100% and the sensitivity is more than 90% for all data with medium and high peptide molar amounts. There is some variation in the accuracy and the sensitivity for the data with low peptide amount samples; the variation can be attributed to low quality spectra obtained due to less abundant peptides. The site assignment appears to be more reliable with HCD data sets than with CID.

### Comparison to other phosphorylation site assignment algorithms

We compare PhosSA with two other widely used site assignment algorithms namely Ascore [17] and PhosphoScore [15]. The first data set that we used for comparison were AQP-2 phosphopeptides: two distinct, doubly-phosphorylated aquaporin-2(AQP2) phosphopeptides, AQP2 peptides phosphorylated at S256/S261 called AQP2-H-(S256/S261) and AQP2 peptides phosphorylated at S256/S269 called AQP2-H-(S256/S269), separately spiked into liver cell lysates, a tissue that does not express AQP2 endogenously [15] were used for comparison. For comparisons we used the recommended parameters for Ascore (threshold of 19) and PhosphoScore (1% D-score). PhosSA surpassed both of these tools in both sensitivity and accuracy as shown in Table 2. The variation in accuracy and sensitivity with varying *dC_n _*exhibited by PhosSA is reported in Additional File 9 (Figure S9).

**Table 2:** Summary of results of phosphorylation site assignment using mass spectra obtained from the analysis of AQP2 peptides.

	AQP2-H-(S256/S261) and AQP2-H-(S256/S269)	
**Algorithm**	**Sensitivity(%)**	**Accuracy(%)**

Ascore	52.4	98.1

PhosphoScore	63.9	92.2

PhosSA	90.9	100.0

PhosSA is designed as a general purpose assignment tool, thus we wanted to test whether PhosSA can deal with a variety of peptides. Therefore, we tested our algorithm on a second data set (provided by Steven Gygi) [15,17] which is derived from mass spectrometry analysis of a library consisting of 380 phosphopeptides (out of which 162 are distinct peptides) from three different families i.e. AS*PXPXAXFEA (Family 1), GAPXPXS*XFEA (Family 2) and ADZZS*STZZFEAK (Family 3) where × is one of the amino acids ADEFGLSTVY and Z is one of the amino acids SDLFGHP. The accuracy and sensitivity results are reported in Table 3 for this data set. As can be seen, PhosSA was able to perform comparable to PhosphoScore in sensitivity and to Ascore in accuracy, combining the best features of the two algorithms. Additional File 10 (Figure S10 (a)) shows the accuracy and sensitivity using PhosSA with varying *dCn *for the phosphopeptide library. With very high thresholds, the sensitivity did not drop below 70% and with very low thresholds the accuracy was always observed to be greater than 85%.

**Table 3:** Summary of results for phosphorylation site assignments using mass spectra obtained from the analysis of Phosphopeptide Library(Ascore> 19, 1%D-score PhosphoScore).

	Phosphopeptide Library	
**Algorithm**	**Sensitivity(%)**	**Accuracy(%)**

Ascore	32.1	99.0

PhosphoScore	76.3	96.6

PhosSA	70.3	98.6

### Assessing the accuracy and sensitivity using a phosphopeptide library

In this section we report the sensitivity and accuracy of the mixed phosphopeptide dataset, described in the previous paragraph, from LC-MS/MS analysis with respect to the individual peptide families with known phosphorylation sites. The three families of peptides described above had different difficulty level for site assignment due to the distance between the actual phosphorylated residue and neighboring potentially phosphorylatable residues. As the distance between the neighbouring potential residues decrease, the number of potential site-determining ions that help PhosSA in deciding the correct sites also decreases, making it more difficult to assign phosphosites. These families are listed in the order of increasing difficultly: AS*PXPXAXFEA, GAPXPXS*XFEA and ADZZS*STZZFEAK where × is one of the amino acids ADEFGLSTVY and Z is one of the amino acids SDLFGHP. The results are summarized in Additional File 10 (Figure S10 (b, c and d)) and can be seen that PhosSA has a high degree of accuracy and sensitivity for all three families. For this data set, Family 3 is considered the most difficult data set for site assignment due to the consecutive order of serines/threonines. Even for Family 3, PhosSA was able to predict sites with 100% accuracy with very high degree of sensitivity. This is in large part due to the inclusion of the redundancy criterion (see methods and Additional File 5 (Figure S5)). The reported results show that PhosSA is capable of accurately assigning phosphorylation sites even for most difficult of peptides and spectra; making it a highly versatile tool for large phosphoproteomics data sets. Large-scale site assignment was also performed using PhosSA for data sets from our previous study [35] consisting of 1737 phosphopeptides. For the pep-tides that passed PhosSA post-processing criteria, 93% of the sites were identical to the sites assigned using Ascore (additional file 11 analysis.xls).

### Assessing the accuracy of phosphorylation site assignment using multiple search engines

PhosSA algorithm was originally designed to take Sequest search results as input [26]. In the current study, we extend PhosSA algorithm to also accept input from the Mascot search engine. In order to make PhosSA compatible with Mascot search results, we developed a converter that could convert Mascot output into compatible PhosSA input. The conversion is done by converting a Proteome Discoverer (.msf) file into a .pepXml file which can be converted into a series of .out files using the given converter.

The Mascot search results for CID and HCD data sets were converted into PhosSA compatible input and the algorithm was executed. The summary of the results are shown in Table 4. As can be seen the accuracy of PhosSA is high for both CID as well as HCD data sets. There are slight differences in the search results for Sequest and Mascot (only the first hit is compared since PhosSA takes the first hit to formulate a potential peptide) that do effect the final site assignment results. For this analysis we have not discarded the site assignments that did not pass the post-processing criteria's. The results obtained for site assignment are very close to the results that we get when using Sequest. Therefore the user should be able to do multiple searches on their datasets and get the site assignments using PhosSA. The comparison of site assignment using Sequest and Mascot are shown in additional file 12 (Comparison-Sequest-Mascot.xls). We plan to extend PhosSA's capability to accept search results from other search engines in the future. The false localization rate (FLR) is also shown in Additional File 13 (Figure S13).

**Table 4:** Summary of PhosSA site assignment results using Mascot for AQP2 mass spectrometry data set using CID and HCD fragmentations (see text for description). N/A means that the AQP2 results were not found in the data set

CID data sets	PhosSA Accuracy(%)	No. of peptides similar to Sequest	No. of peptides different than Sequest
AQP2-H-(S256/S261)	94.6	690	17

AQP2-M-(S256/S261)	94.4	714	26

AQP2-L-(S256/S261)	100	665	26

AQP2-H-(S264)	100	658	9

AQP2-M-(S264)	89	661	24

AQP2-L-(S264)	60	801	17

HCD data sets			

AQP2-H-(S256/S261)	94.5	857	13

AQP2-M-(S256/S261)	95.9	921	25

AQP2-L-(S256/S261)	100	992	27

AQP2-H-(S264)	100	822	14

AQP2-M-(S264)	96.6	804	20

AQP2-L-(S264)	75	935	12

### Execution time

**Figure 5:**
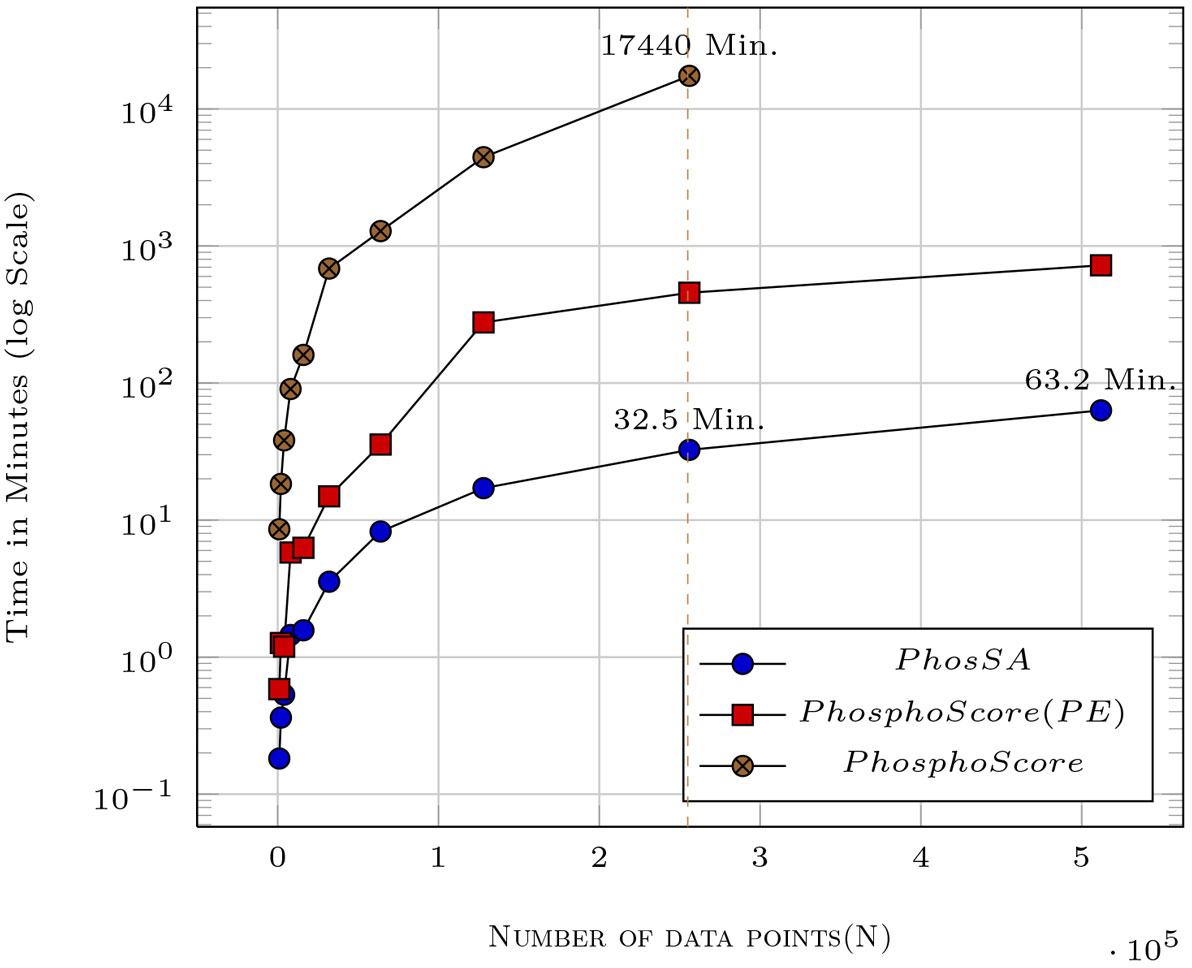
**Execution times with increasing number of spectra**. The time taken by PhosSA for 2.5 *× *10^5 ^spectra is just 32.5 minutes whereas for PhosphoScore with parameter estimation (PhosphoScore(PE)) it takes 17740 minutes. The execution time for PhosphoScore without any parameter estimation is also shown. It must be noted that parameter estimation is required for each new dataset analyzed using PhosphoScore.

The complexity analysis suggests that PhosSA running time should increase linearly with increase in the number of spectra. Although, the algorithm gives theoretical guarantees on the running times, the observed running times depend on the implementation. Experimental validation is necessary in order to determine if he observed running times are consistent with the theoretical analysis. In Figure 5, we report results for up to *0.5 million *peptides from replicated HCD data sets using our algorithm. The task of assigning sites for 0.5 million spectra was accomplished in just 63.2 minutes when using our compute server with modest hardware whereas PhosphoScore (with and without parameter estimation) was substantially slower. The linearly increasing running time with increasing peptides/spectra, makes PhosSA an ideal tool for processing large amounts of phosphoproteomics datasets from state-of-the-art mass spectrometers. High performance algorithms are shown to be highly successful in analysing large-scale genomics and proteomics data sets and reduces execution time significantly [36,37]. We have also introduced a parallel implementation of the PhosSA algorithm using multicore machines in [38] which further decreases the computational resources required for assignments.

### Graphical User Interface (GUI) and input/output formats

The implementation of the PhosSA algorithm is available as a graphical user interface(GUI). The implemented GUI consists of two parts; 1) the upper portion of the GUI is related to conversion of various search engine formats such as Sequest and Mascot into PhosSA compatible inputs; 2) the lower portion of the GUI allows user to select the .dta and .out files that are used to process site assignments. The user can also select fragmentation method and the *dC_n _*threshold. A snapshot of the GUI is shown in Additional File 14 (Figure S14).

After the PhosSA execution on the selected data set, the results are written in a file named "outresult2". The result files consists of 5 columns and a snapshot of the results file is shown in Additional File 15 (Figure S15). Column A shows the scan number of the peptide, B shows the peptide with the assignment, C shows the *dC_n _*of the peptide, D shows the redundancy metric and column E shows the final verdict (passed or ambiguous) given by PhosSA. There are multiple reason why the algorithm would assign a phosphopeptide as "ambiguous" e.g. poor spectral quality, the presence of composite spectra (i.e. spectra containing more than one distinct peptide species), and instances where multiple phosphorylatable residues are in close proximity (resulting in a low *dCn *value). In cases where the phosphopeptide is reported "ambiguous", we suggest users may try an additional program CPhos [39], to break the tie between assignment(s). CPhos utilizes an information-theory based algorithm to assess the conservation of phosphorylation sites among species. We assert that the site (even if it is reported ambiguous by PhosSA) is likely to be correctly assigned if it is well conserved across multiple species, as conserved phosphorylation sites are more likely to play functional roles than non-conserved sites [40,41]. High accuracy clustering techniques for mass spectrometry data can also mitigate the effects of low S/N ratio spectra [42,43] to further reduce the number of ambiguous assignments. The GUI is freely available for non-commercial use.

## Discussion

In this paper we report the design and implementation of the first dynamic programming solution for phosphorylation site assignment problem of mass spectrometry data. The designed framework allowed us to develop a highly accurate and scalable solution for large MS data sets. In the paper we describe the design details of the algorithm, its space and time complexity analysis, experiments performed to evaluate the algorithm, and running times of the program with increasing number of spectra. The implemented algorithm, referred to as PhosSA, assigns a single score to each phosphopeptide which is then post-processed using our quality control criteria. A rigorous quality assessment of the results from PhosSA was done using experimental mass spectrometry data using peptides with known phosphorylation sites with varying characteristics such as the position of the sites, the peptide amounts in the samples and CID/HCD fragmentation methodologies. For the experiments we conducted using real phosphoproteomics data sets, PhosSA was able to carry out site assignment tasks with high accuracy (close to 100%) and sensitivity (around 90%) in a rapid fashion (approx. 0.5 millon spectra per hour). The presence of consecutive phosphorylatable residues in peptides are the most difficult to assign due to limited number of site determining peaks. Our experiments suggest that PhosSA was able to assign correct sites even for these spectra (Family 3). We also report that PhosSA was able to do better in terms of accuracy as well as sensitivity when compared to other tools such as *Ascore*, *PhosphoScore *and *PhosphoRS*. Unlike Ascore and PhosphoScore, PhosSA is able to deal with both HCD data sets as well as iTRAQ- or SILAC-labelled CID data sets as demonstrated in [44]. One of the pitfalls of most proteomics tools is the inability to deal with different file formats. The current implementation uses the Sequest (.dta and .out) search results as well as Mascot results from Proteome Discoverer (.msf). In order to assist users we have also included a .pepxml to .out format convertor, which will allow users to utilize other search tools (Inspect, OMSSA) that can be used in conjunction with PhosSA. PhosSA has been designed, optimized and tested for phosphoproteomics data but the design of the generic framework can be readily adapted for analysis of other post-translations modifications and is an interesting area for future research. Currently, our implementation requires a separate GUI for accepting Sequest and Mascot search results. The future plans include integrating the different GUI's into a single framework that would allow users to use one GUI for all search engines.

## Competing interests

The authors declare that they have no competing interests.

## Authors' Contributions

Fahad Saeed conceived, designed & implemented the algorithm, designed & performed quality experiments, designed & studied the scalability of the algorithm and wrote the manuscript. Trairak Pisitkun and Jason D. Hoffert helped design the algorithm and performed experiments to generate experimental mass spectrometry data. They also helped in writing of the manuscript. Sara Rashidian implemented part of the graphical user interface and related conversion tools for public use. Guanghui Wang and Marjan Gucek produced mass spectrometry data in Proteomics Core Facility at System Biology Center, NHLBI, NIH. Mark A. Knepper helped in design of the experiments and writing of the manuscript.

## Supplementary Material

Additional file 1 **Figure S1**. Effects of fragmentation ions on CID data sets. The effects of including the following fragmentation ions in PhosSA algorithm is shown in the figure: (b/y) with neutral losses of phosphoric acid (denoted by P-only), (b/y) with neutral losses of water and ammonia (denoted by water and ammonia), (b/y) with a neutral loss of phosphoric acid and water (denoted by -P-water) and (b/y) with a neutral loss of phosphoric acid and ammonia (denoted by P-Ammonia). The *Threshold *in this figure is defined as Threshold = (Peak intensity)÷(Maximum peak intensity). Only the peaks that pass the Threshold criterion are considered. Click here for file

Additional file 2 **Figure S2**. Effects of fragmentation ions on HCD data sets is shown The effects of including the following fragmentation ions in PhosSA algorithm is shown in the figure: (b/y) with neutral losses of phosphoric acid (denoted by P-only), (b/y) with neutral losses of water and ammonia (denoted by water and ammonia), (b/y) with a neutral loss of phosphoric acid and water (denoted by -P-water) and (b/y) with a neutral loss of phosphoric acid and ammonia (denoted by P-Ammonia). The *Threshold *in this figure is defined as Threshold = (Peak intensity)÷(Maximum peak intensity). Only the peaks that pass the Threshold criterion are considered. Click here for file

Additional file 3 **Figure S3**. An *M S^2 ^*spectrum of a peptide. The peaks matched to two or more theoretical fragment ions as depicted in red circles. Click here for file

Additional file 4 **Figure S4**. The effect of window size (*ζ*) on the accuracy and sensitivity of the results obtained by executing PhosSA on the phosphopeptide library. Click here for file

Additional file 5 **Figure S5**. Accuracy and sensitivity with no redundancy criterion and no parameter optimization, for peptide Family1=AS*PXPXAXFEA, Family2=GAPXPXS*XFEA, Family3=ADZZS*STZZFEAK where × is one of the amino acids ADEFGLSTVY and Z was one of the amino acids SDLFGHP with varying (*dCn*) threshold is shown. The overall accuracy and sensitivity of the whole data set, consisting of Family1, Family2 and Family3, is also shown. Click here for file

Additional file 6 **Figure S6**. The limitation of using *dC_n _*threshold criterion alone is shown. The figure shows the results from multiple spectra of a peptide with a known phosphorylation site (indicated by asterisks) correctly assigned using our dynamic programming algorithm. The peptides in red have smaller *dC_n _*that have not passed the threshold of 0.9, although they have been correctly assigned by the dynamic programming module. The addition of redundancy, an additional criterion to take into account, eliminates this limitation and peptides marked in red pass the post-processing criteria. Click here for file

Additional file 7 **Figure S7**. With the number of times a phosphorylation site is assigned using dynamic programming, the probability of that site being incorrect decreases sharply because of the multiplicative factor of probability (P = 0.5 for each assigned site for a two potential phosphorylation sites). The effect of varying redundancy threshold is shown. Click here for file

Additional file 8 **Figure S8**. The collision energy optimization for optimal HCD fragmentation of phosphopeptides is shown. CE(%) denotes the percentage of collision energy used; # pep hits is the total number of peptide-spectrum-match identified and # of quantifiable denotes the number of spectra that have iTRAQ reporter ions present for quantification. Click here for file

Additional file 9 **Figure S9**. Accuracy(Acc.) and Sensitivity(Sens.) trend for AQP2-H-(S256/S261)(denoted by S7) and AQP2-H- (S256/S269) (denoted by S8) AQP2 phosphorylated at 256 and 261 (RQS*VELHS*PQSLPRGSK) and at 256 and 269 (QS*VELHSPQSLPRGS*K) respectively with varying (*dCn*) threshold. Click here for file

Additional file 10 **Figure S10**. The accuracy and sensitivity of site assignment for the phosphopeptide library with varying (*dCn*) thresholds analyzed by PhosSA; A, all peptides; B, Family1=AS*PXPXAXFEA; C, Family2=GAPXPXS*XFEA; D, Family3=ADZZS*STZZFEAK; × is one of the amino acids ADEFGLSTVY and Z was one of the amino acids SDLFGHP. Click here for file

Additional file 11 **Analysis File of Ascore and PhosSA**. Excel file is included that does a head-to-head comparison of Ascore and PhosSA using our previously published large-scale data set. (Analysis.xls) Click here for file

Additional file 12 **Analysis File of PhosSA results using Sequest and Mascot**. Excel file is included that does a head-to-head comparison of PhosSA results using Sequest and Mascot search engines on the same raw CID and HCD data(Comparison-Sequest-Mascot.xls) Click here for file

Additional file 13 **Figure S13**. The sensitivity defined as TP/(TP+FN) where TP=True Positive and FN=False Negative is plotted against the False Localization Rate (FLR)% for phosphopeptide library. Click here for file

Additional file 14 **Figure S14**. Graphical User Interface developed for phosphorylation site assignment is shown. Click here for file

Additional file 15 **Figure S15**. The output format in a text file is shown. Click here for file

## Declarations

This work and the publication cost for this manuscript was funded by the operating budget of Division of Intramural Research, National Heart, Lung and Blood Institute, National Institutes of Health (NIH), Project ZO1-HL001285.

This article has been published as part of *Proteome Science *Volume 11 Supplement 1, 2013: Selected articles from the IEEE International Conference on Bioinformatics and Biomedicine 2012: Proteome Science. The full contents of the supplement are available online at http://www.proteomesci.com/supplements/11/S1.
