# Supplementary figures and images for: PhosSA: Fast and accurate phosphorylation site assignment algorithm for mass spectrometry data
Source: Proteome Sci. 2013 Nov 7;11(Suppl 1):S14. doi: 10.1186/1477-5956-11-S1-S14 (PMC3909108; doi:10.1186/1477-5956-11-S1-S14)

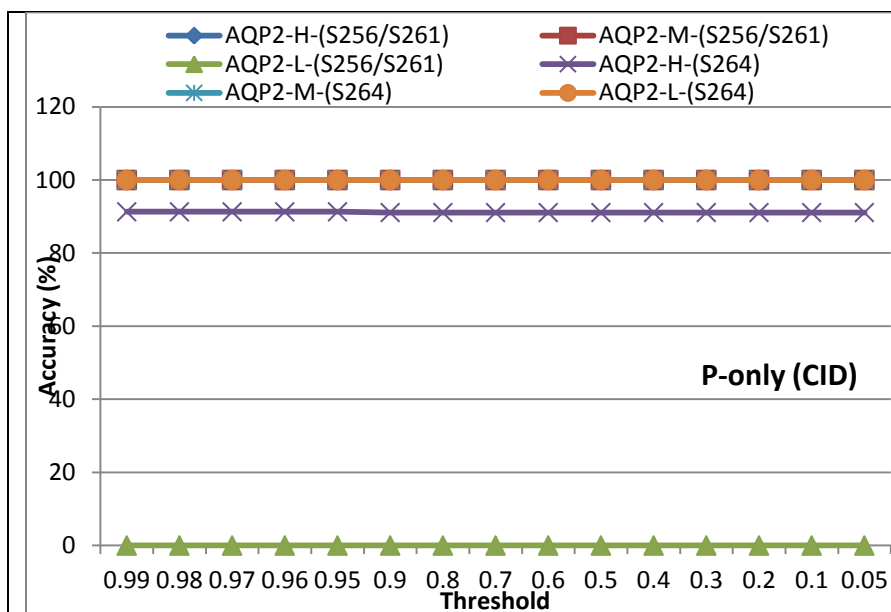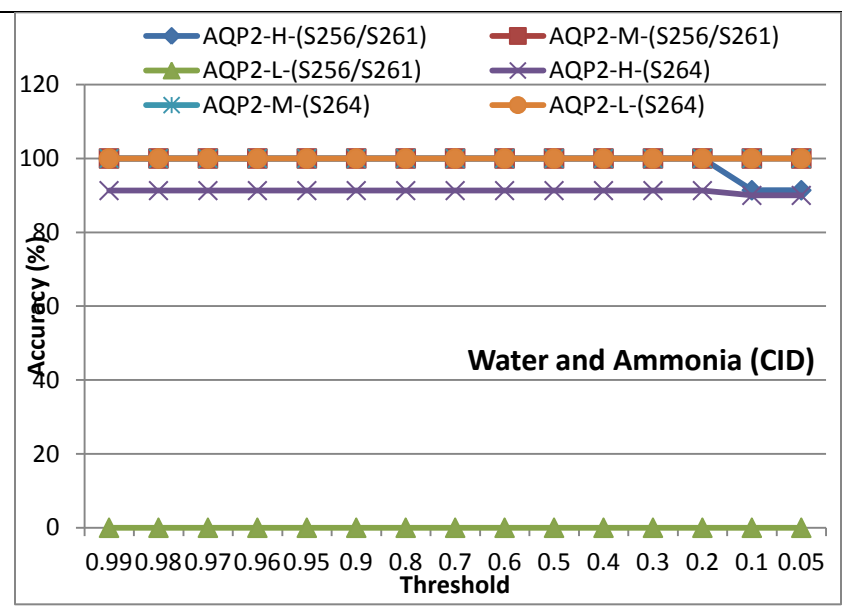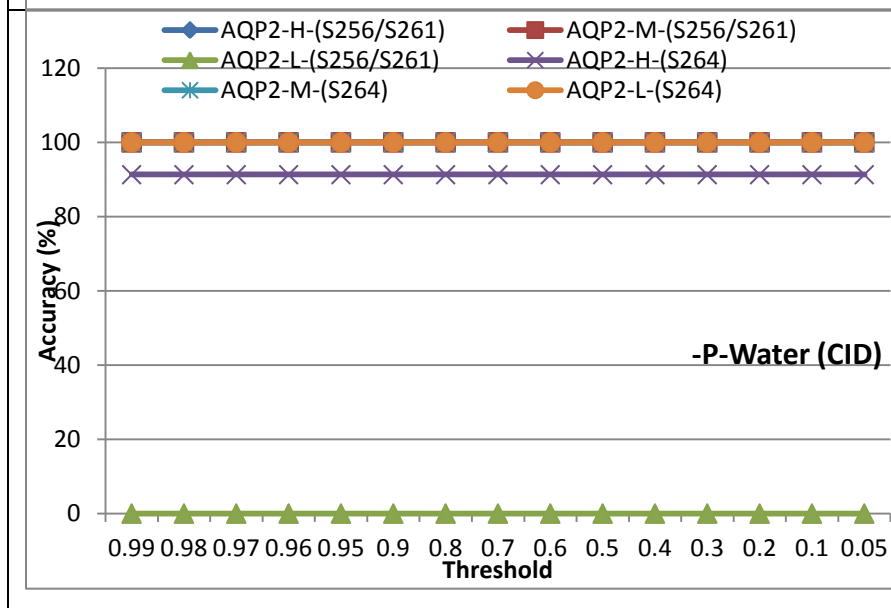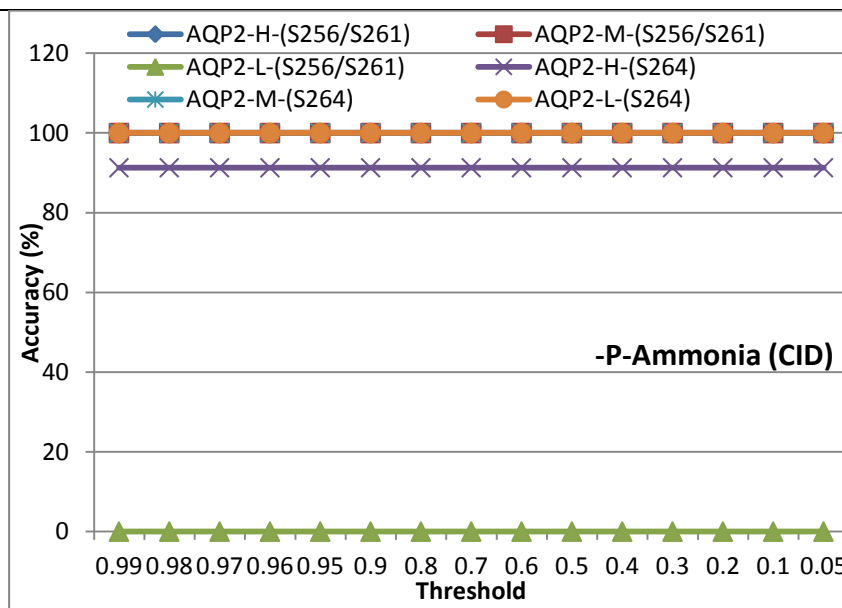

Supplement: Additional file 1 — Figure S1. Effects of fragmentation ions on CID data sets. The effects of including the following fragmentation ions in PhosSA algorithm is shown in the figure: (b/y) with neutral losses of phosphoric acid (denoted by P-only), (b/y) with neutral losses of water and ammonia (denoted by water and ammonia), (b/y) with a neutral loss of phosphoric acid and water (denoted by -P-water) and (b/y) with a neutral loss of phosphoric acid and ammonia (denoted by P-Ammonia). The Threshold in this figure is defined as Threshold = (Peak intensity)÷(Maximum peak intensity). Only the peaks that pass the Threshold criterion are considered. [file 1477-5956-11-S1-S14-S1.pdf]

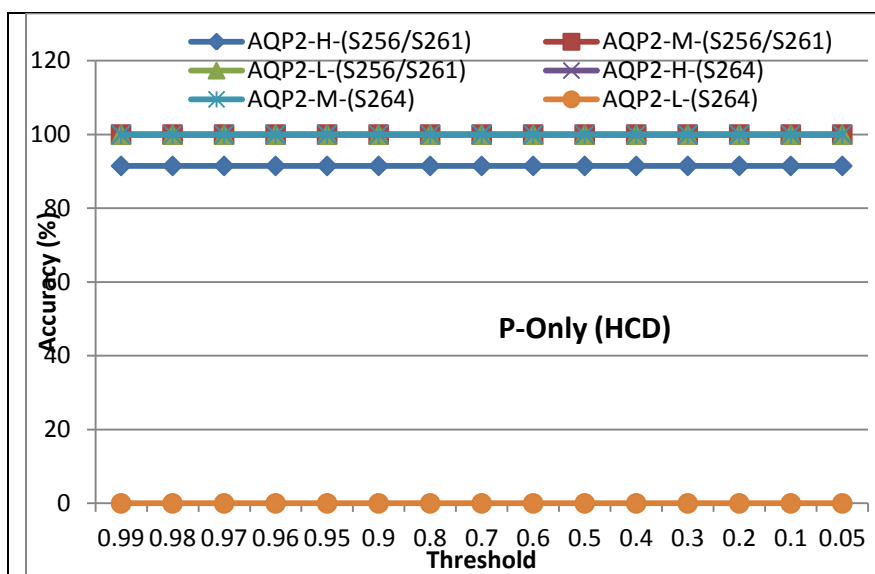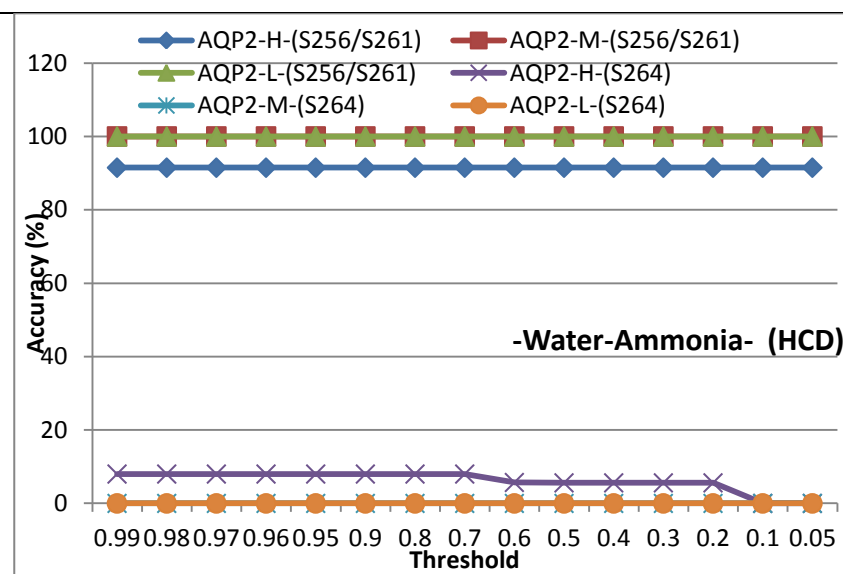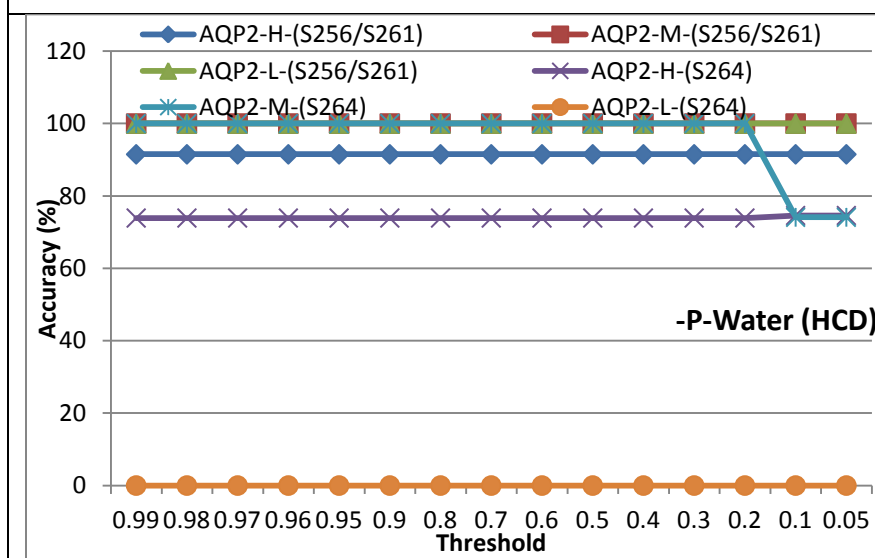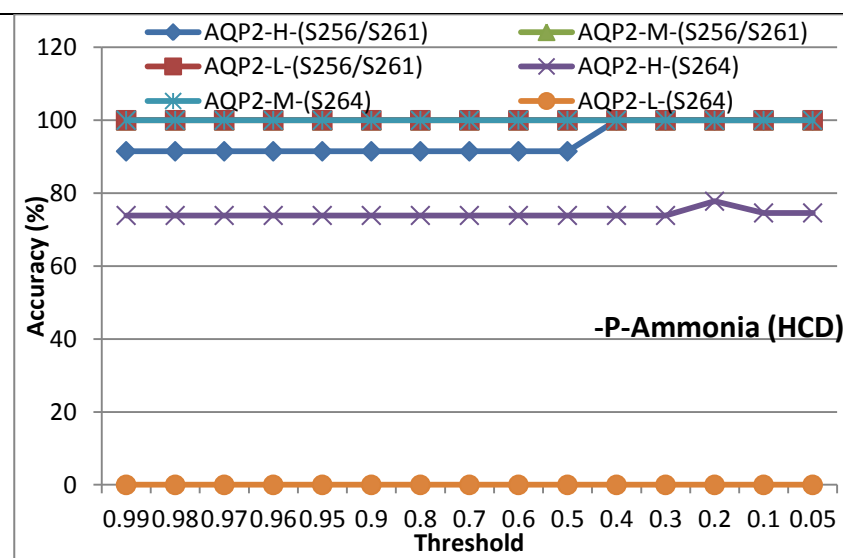

Supplement: Additional file 2 — Figure S2. Effects of fragmentation ions on HCD data sets is shown The effects of including the following fragmentation ions in PhosSA algorithm is shown in the figure: (b/y) with neutral losses of phosphoric acid (denoted by P-only), (b/y) with neutral losses of water and ammonia (denoted by water and ammonia), (b/y) with a neutral loss of phosphoric acid and water (denoted by -P-water) and (b/y) with a neutral loss of phosphoric acid and ammonia (denoted by P-Ammonia). The Threshold in this figure is defined as Threshold = (Peak intensity)÷(Maximum peak intensity). Only the peaks that pass the Threshold criterion are considered. [file 1477-5956-11-S1-S14-S2.pdf]

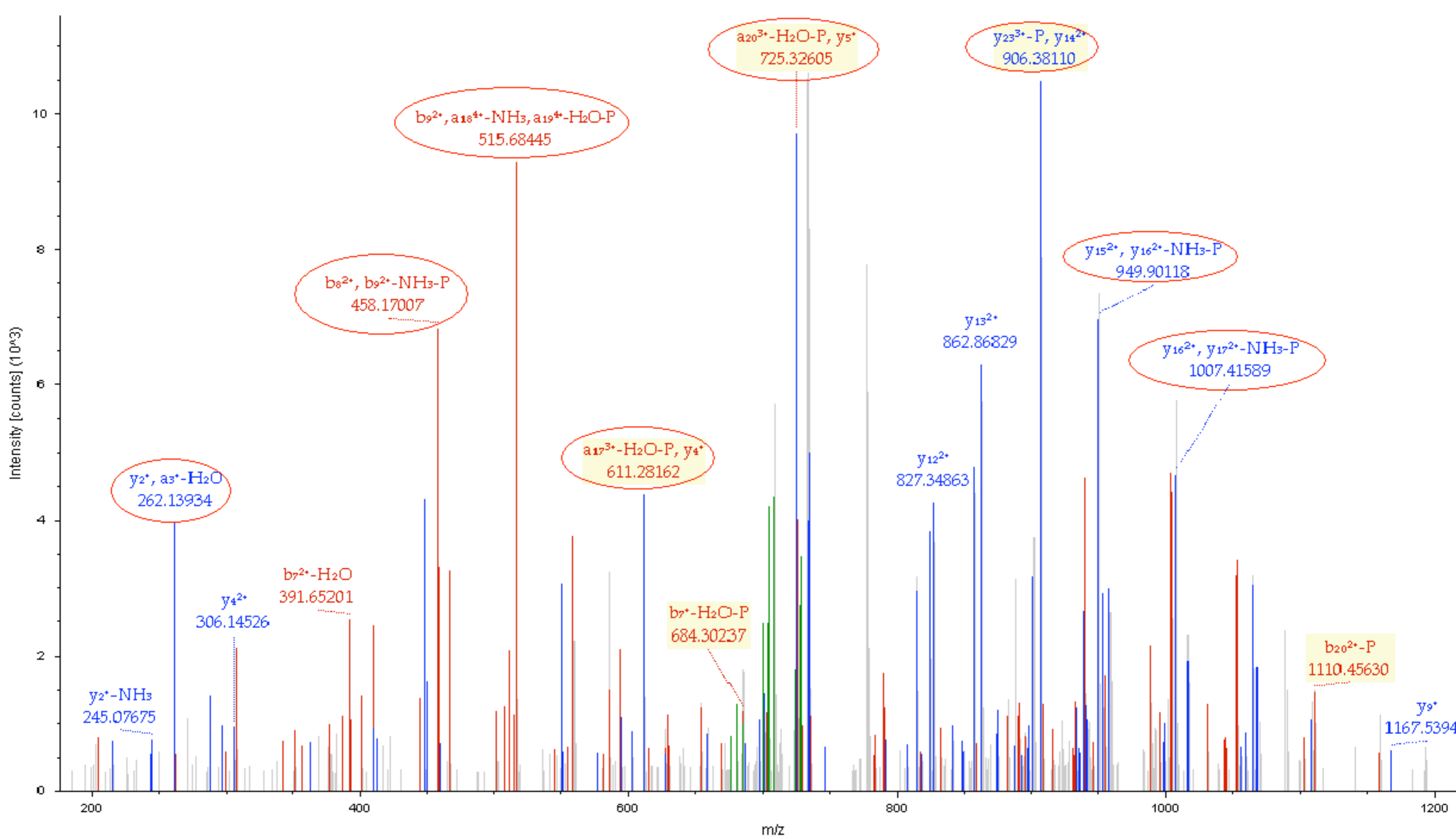

Supplement: Additional file 3 — Figure S3. An M S2 spectrum of a peptide. The peaks matched to two or more theoretical fragment ions as depicted in red circles. [file 1477-5956-11-S1-S14-S3.pdf]

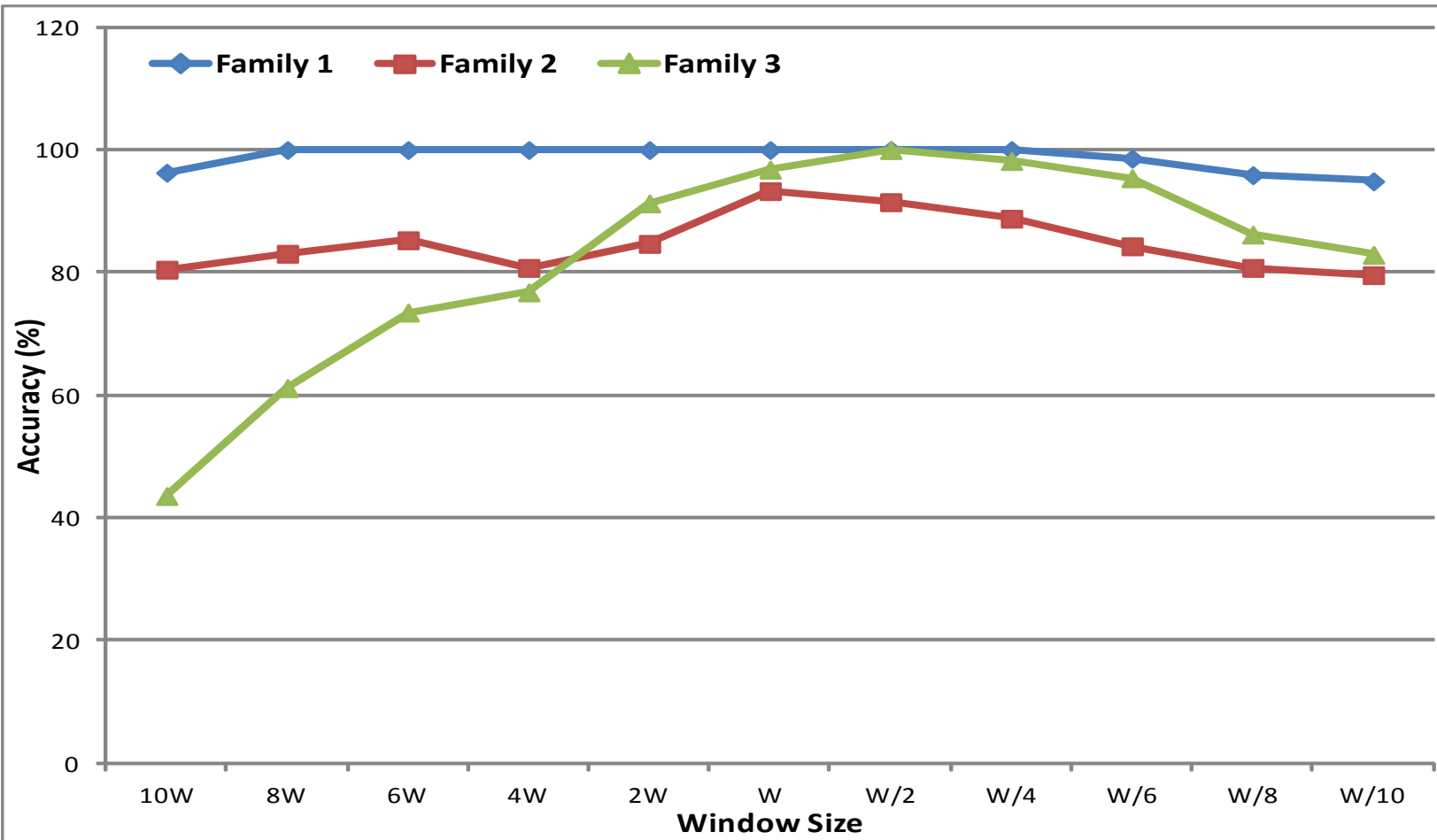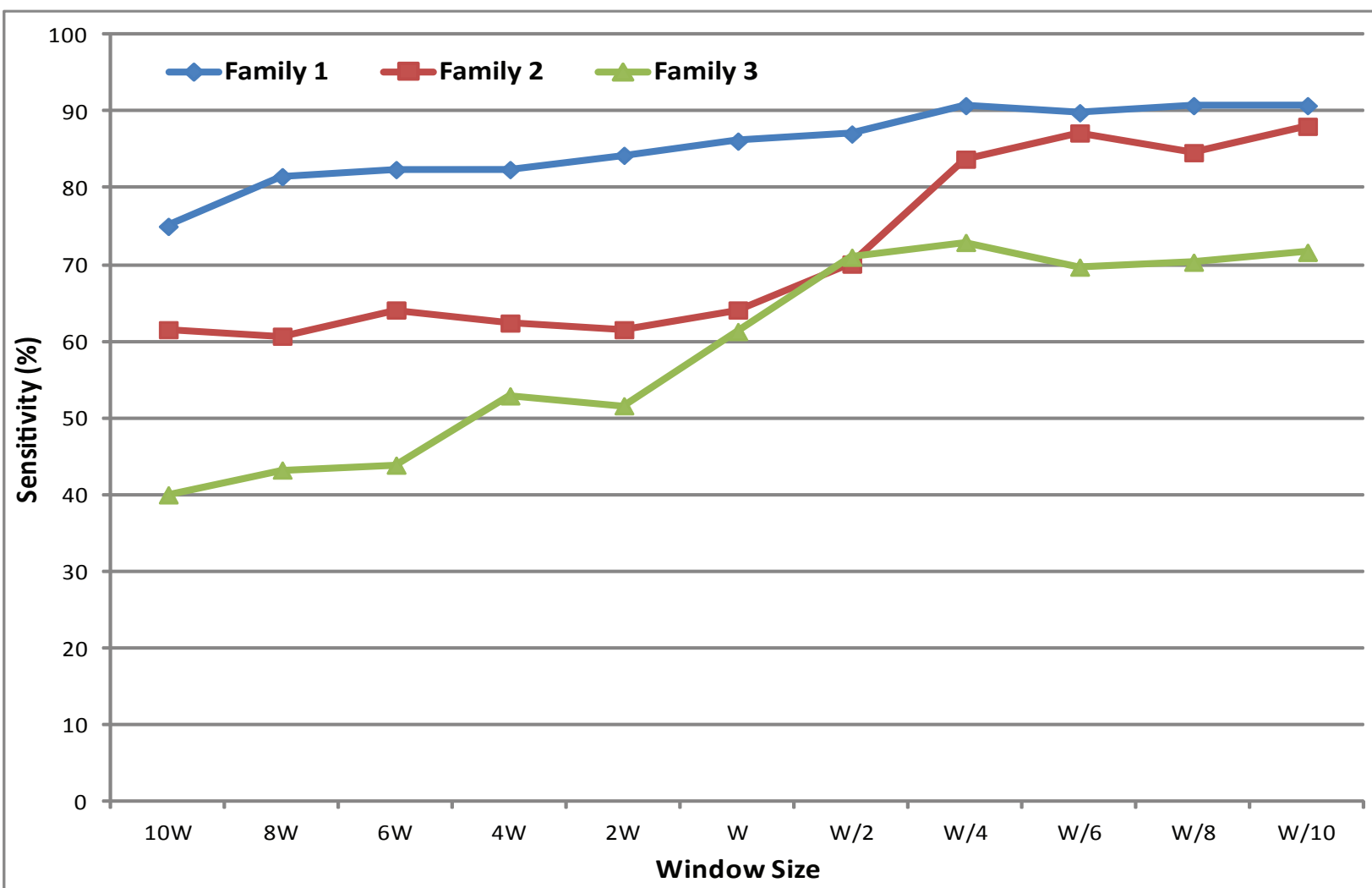

Supplement: Additional file 4 — Figure S4. The effect of window size (ζ) on the accuracy and sensitivity of the results obtained by executing PhosSA on the phosphopeptide library. [file 1477-5956-11-S1-S14-S4.pdf]

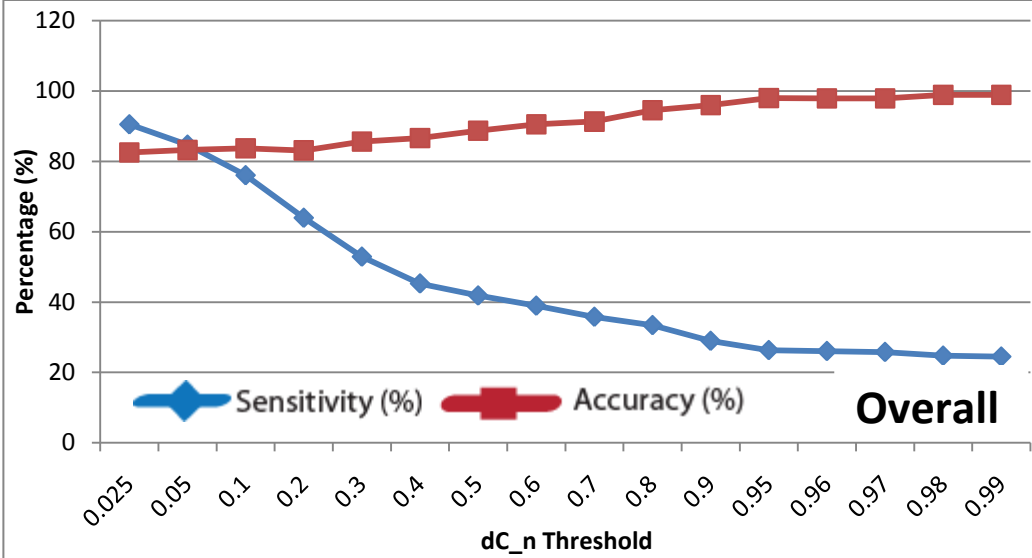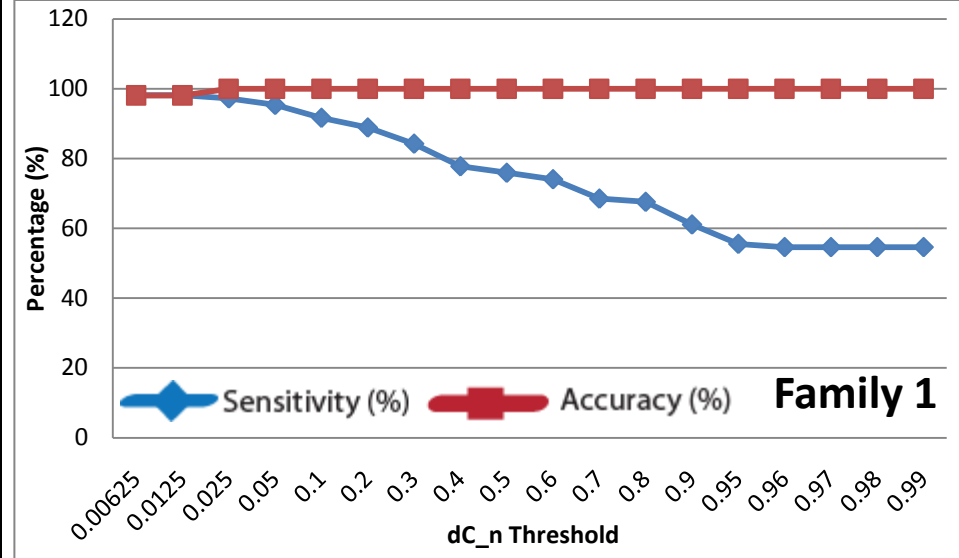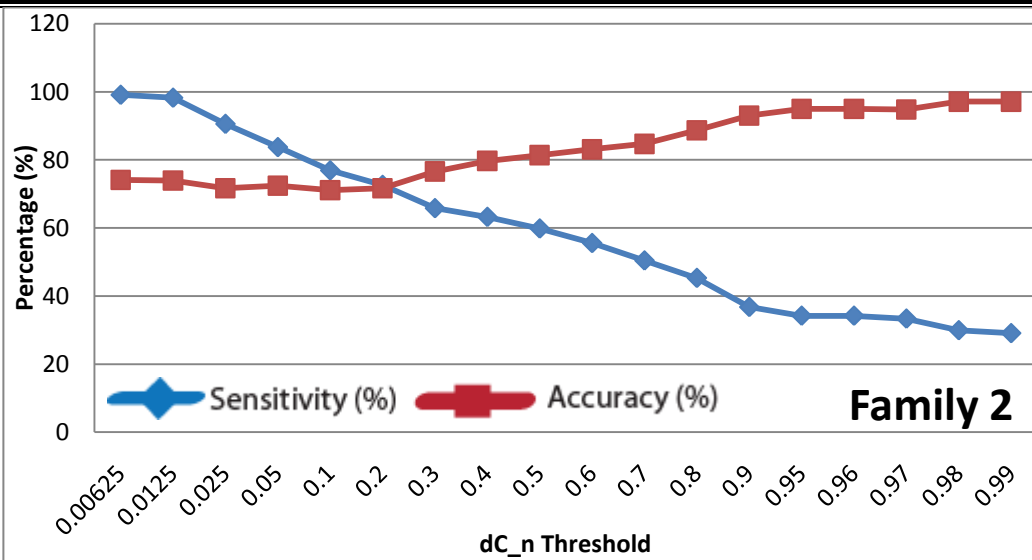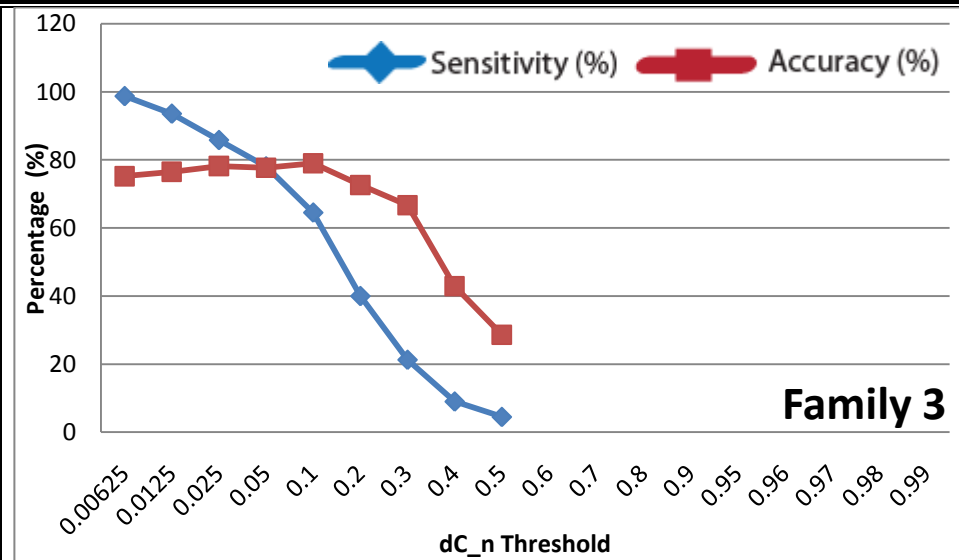

Supplement: Additional file 5 — Figure S5. Accuracy and sensitivity with no redundancy criterion and no parameter optimization, for peptide Family1=AS*PXPXAXFEA, Family2=GAPXPXS*XFEA, Family3=ADZZS*STZZFEAK where × is one of the amino acids ADEFGLSTVY and Z was one of the amino acids SDLFGHP with varying (dCn) threshold is shown. The overall accuracy and sensitivity of the whole data set, consisting of Family1, Family2 and Family3, is also shown. [file 1477-5956-11-S1-S14-S5.pdf]

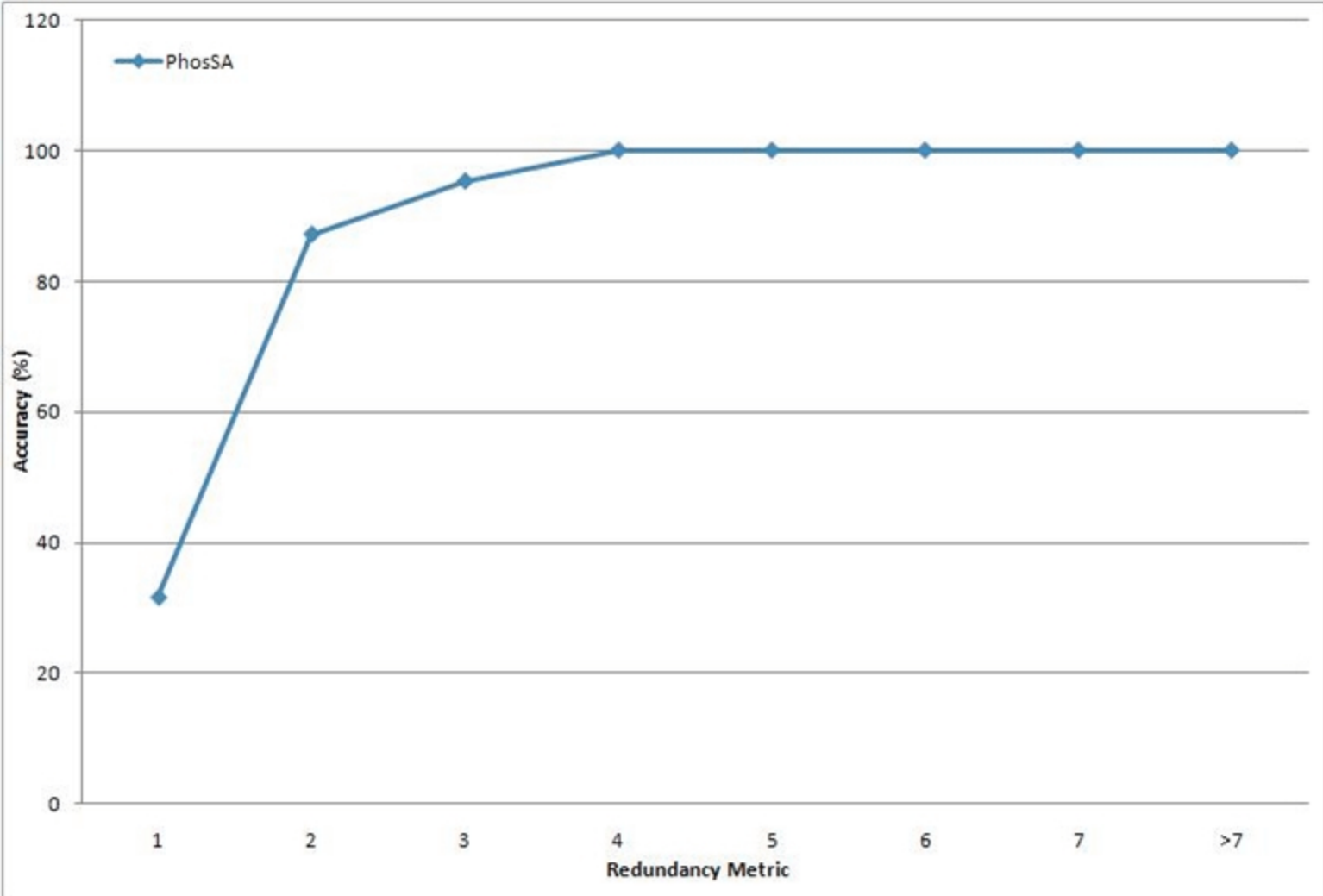

Supplement: Additional file 7 — Figure S7. With the number of times a phosphorylation site is assigned using dynamic programming, the probability of that site being incorrect decreases sharply because of the multiplicative factor of probability (P = 0.5 for each assigned site for a two potential phosphorylation sites). The effect of varying redundancy threshold is shown. [file 1477-5956-11-S1-S14-S7.pdf]

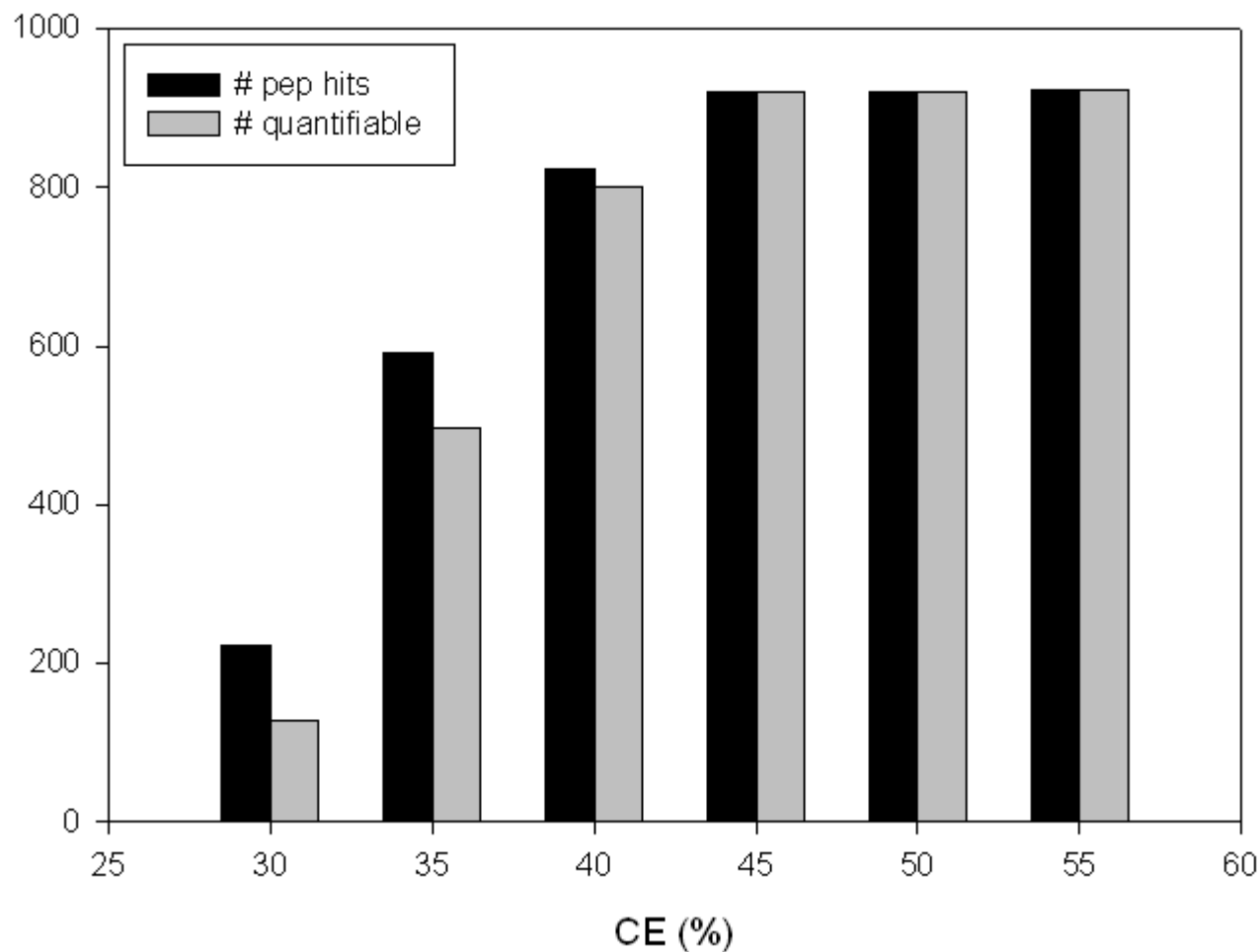

Supplement: Additional file 8 — Figure S8. The collision energy optimization for optimal HCD fragmentation of phosphopeptides is shown. CE(%) denotes the percentage of collision energy used; # pep hits is the total number of peptide-spectrum-match identified and # of quantifiable denotes the number of spectra that have iTRAQ reporter ions present for quantification. [file 1477-5956-11-S1-S14-S8.pdf]

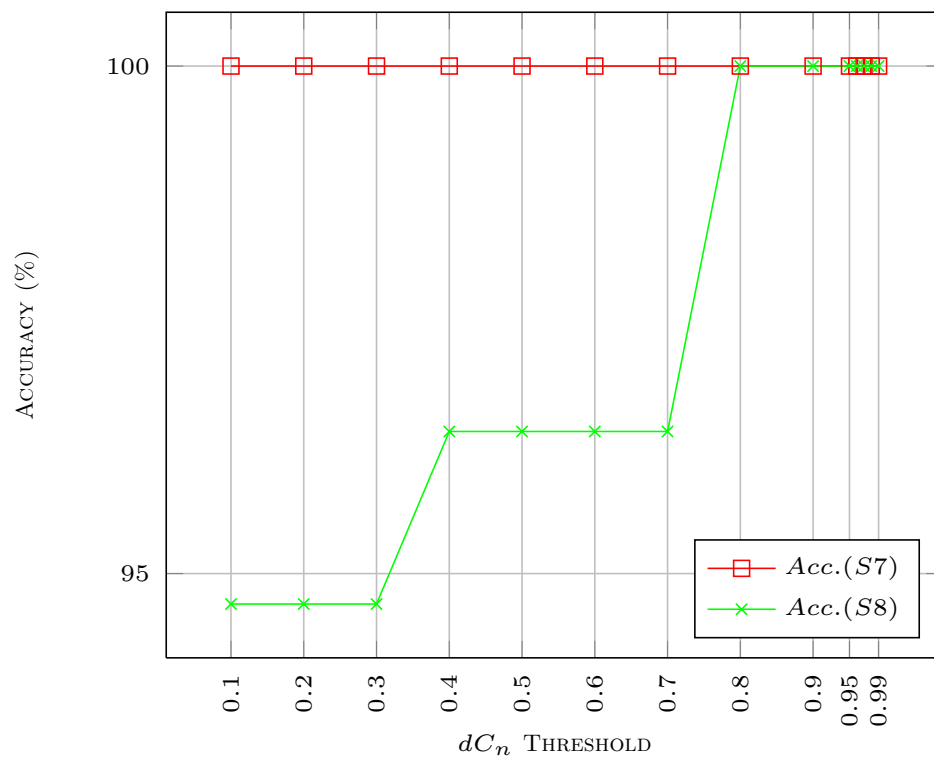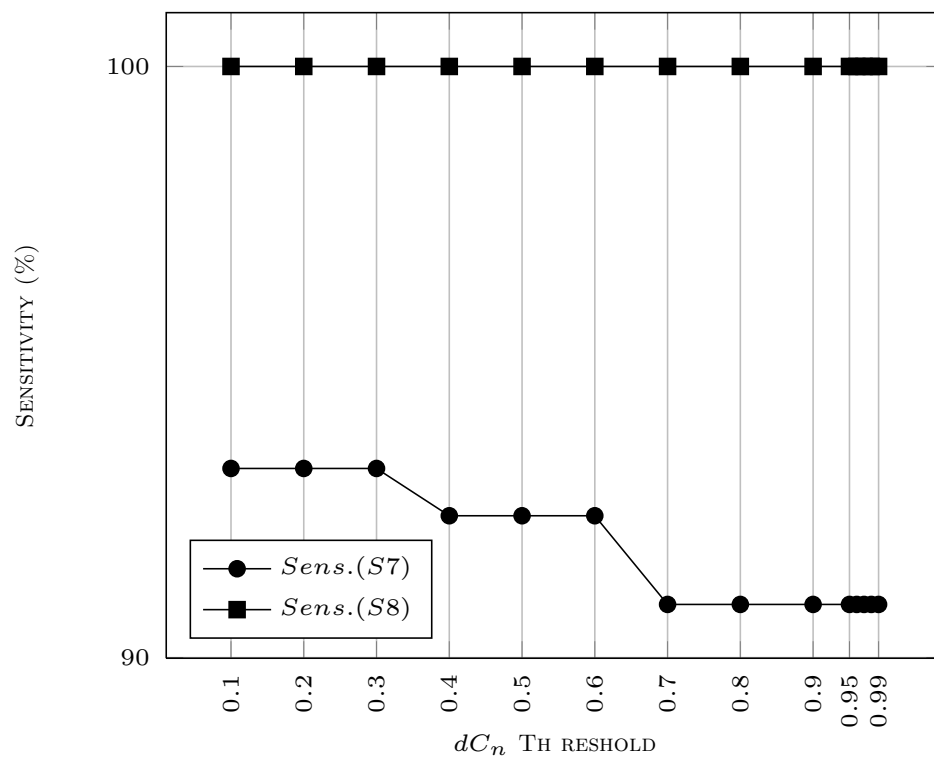

Supplement: Additional file 9 — Figure S9. Accuracy(Acc.) and Sensitivity(Sens.) trend for AQP2-H-(S256/S261)(denoted by S7) and AQP2-H- (S256/S269) (denoted by S8) AQP2 phosphorylated at 256 and 261 (RQS*VELHS*PQSLPRGSK) and at 256 and 269 (QS*VELHSPQSLPRGS*K) respectively with varying (dCn) threshold. [file 1477-5956-11-S1-S14-S9.pdf]

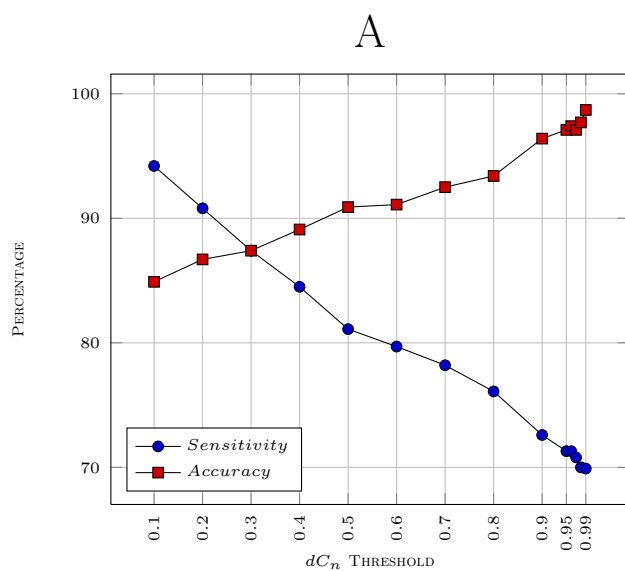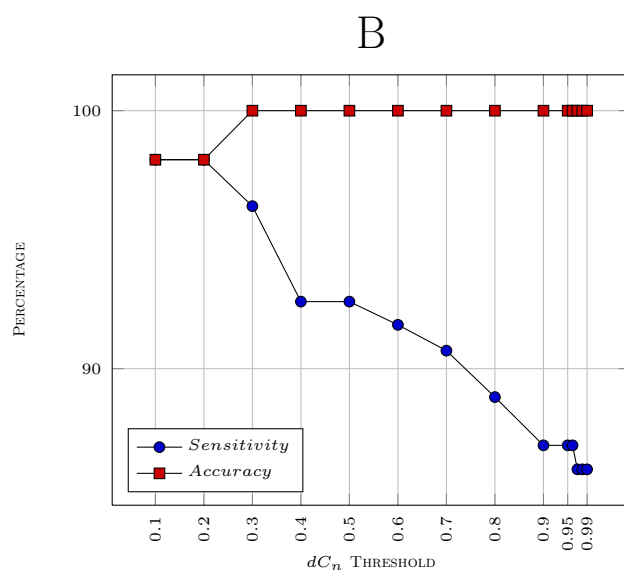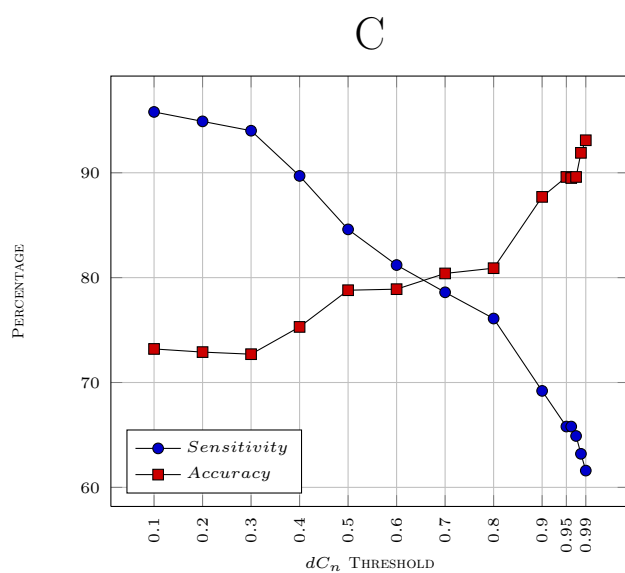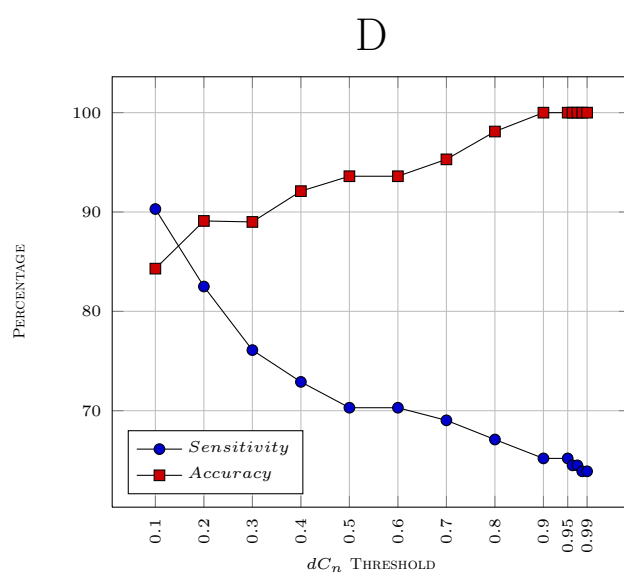

Supplement: Additional file 10 — Figure S10. The accuracy and sensitivity of site assignment for the phosphopeptide library with varying (dCn) thresholds analyzed by PhosSA; A, all peptides; B, Family1=AS*PXPXAXFEA; C, Family2=GAPXPXS*XFEA; D, Family3=ADZZS*STZZFEAK; × is one of the amino acids ADEFGLSTVY and Z was one of the amino acids SDLFGHP. [file 1477-5956-11-S1-S14-S10.pdf]

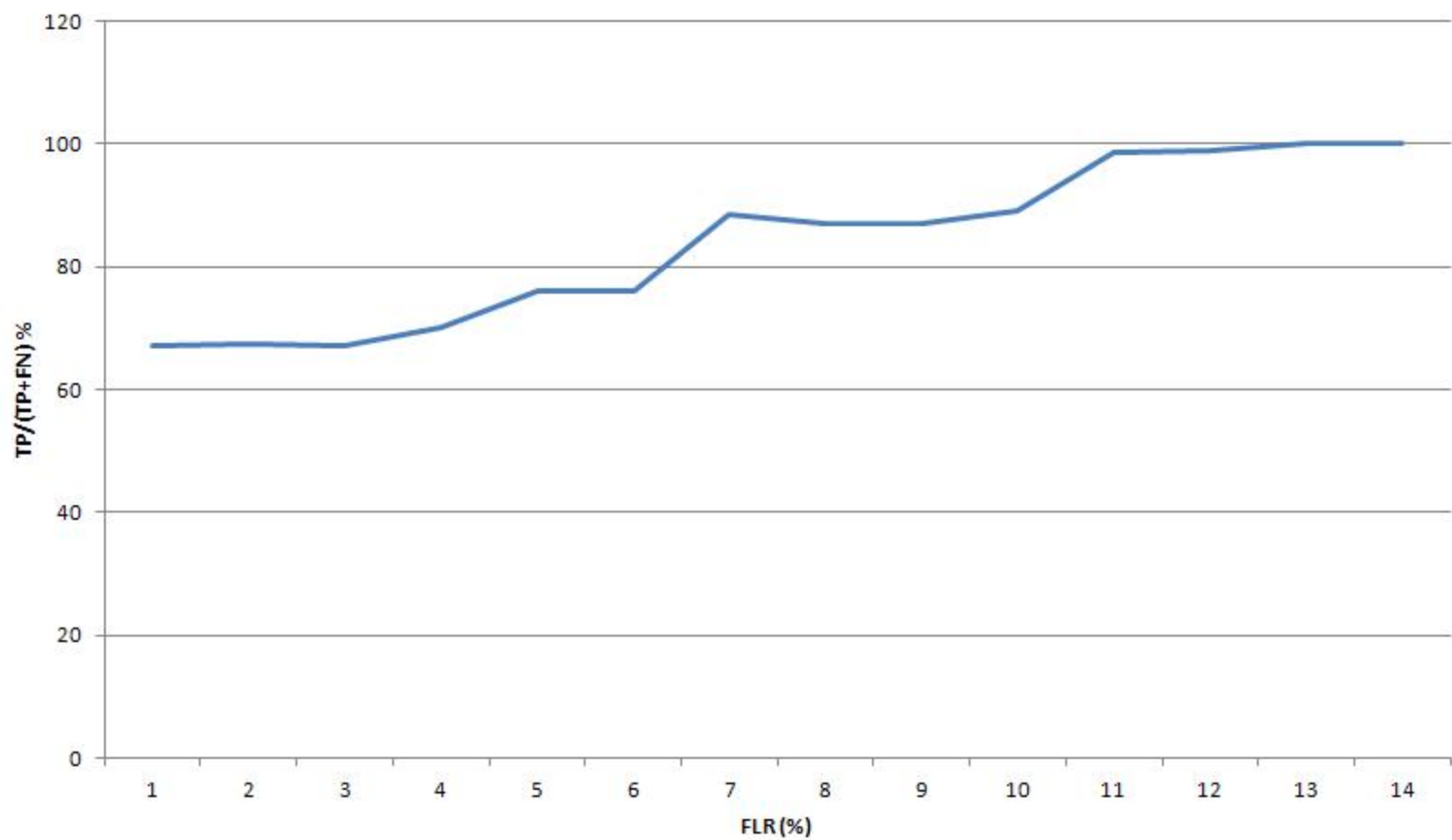

Supplement: Additional file 13 — Figure S13. The sensitivity defined as TP/(TP+FN) where TP=True Positive and FN=False Negative is plotted against the False Localization Rate (FLR)% for phosphopeptide library. [file 1477-5956-11-S1-S14-S13.pdf]
