# Supplementary material for: PhosSA: Fast and accurate phosphorylation site assignment algorithm for mass spectrometry data
Source: Proteome Sci. 2013 Nov 7;11(Suppl 1):S14. doi: 10.1186/1477-5956-11-S1-S14 (PMC3909108; doi:10.1186/1477-5956-11-S1-S14)
Supplement: Additional file 6 — Figure S6. The limitation of using dCn threshold criterion alone is shown. The figure shows the results from multiple spectra of a peptide with a known phosphorylation site (indicated by asterisks) correctly assigned using our dynamic programming algorithm. The peptides in red have smaller dCn that have not passed the threshold of 0.9, although they have been correctly assigned by the dynamic programming module. The addition of redundancy, an additional criterion to take into account, eliminates this limitation and peptides marked in red pass the post-processing criteria. [file 1477-5956-11-S1-S14-S6.pdf]

**Assigned Peptides**	***d*C_n Threshold**
R.RSS*VFAAEIMDAFDR.S	0.949384818
R.RSS*VFAAEIMDAFDR.S	0.927935122
R.RSS*VFAAEIMDAFDR.S	0.944661598
R.RSS*VFAAEIMDAFDR.S	1
R.RSS*VFAAEIMDAFDR.S	0.948100257
R.RSS*VFAAEIMDAFDR.S	1
R.RSS*VFAAEIMDAFDR.S	0.69411405
R.RSS*VFAAEIMDAFDR.S	0.362478778
R.RSS*VFAAEIMDAFDR.S	1
R.RSS*VFAAEIMDAFDR.S	1
R.RSS*VFAAEIMDAFDR.S	1
R.RSS*VFAAEIMDAFDR.S	0.950292556
R.RSS*VFAAEIMDAFDR.S	1
