# Supplementary material for: PhosSA: Fast and accurate phosphorylation site assignment algorithm for mass spectrometry data
Source: Proteome Sci. 2013 Nov 7;11(Suppl 1):S14. doi: 10.1186/1477-5956-11-S1-S14 (PMC3909108; doi:10.1186/1477-5956-11-S1-S14)
Supplement: Additional file 14 — Figure S14. Graphical User Interface developed for phosphorylation site assignment is shown. [file 1477-5956-11-S1-S14-S14.pdf]

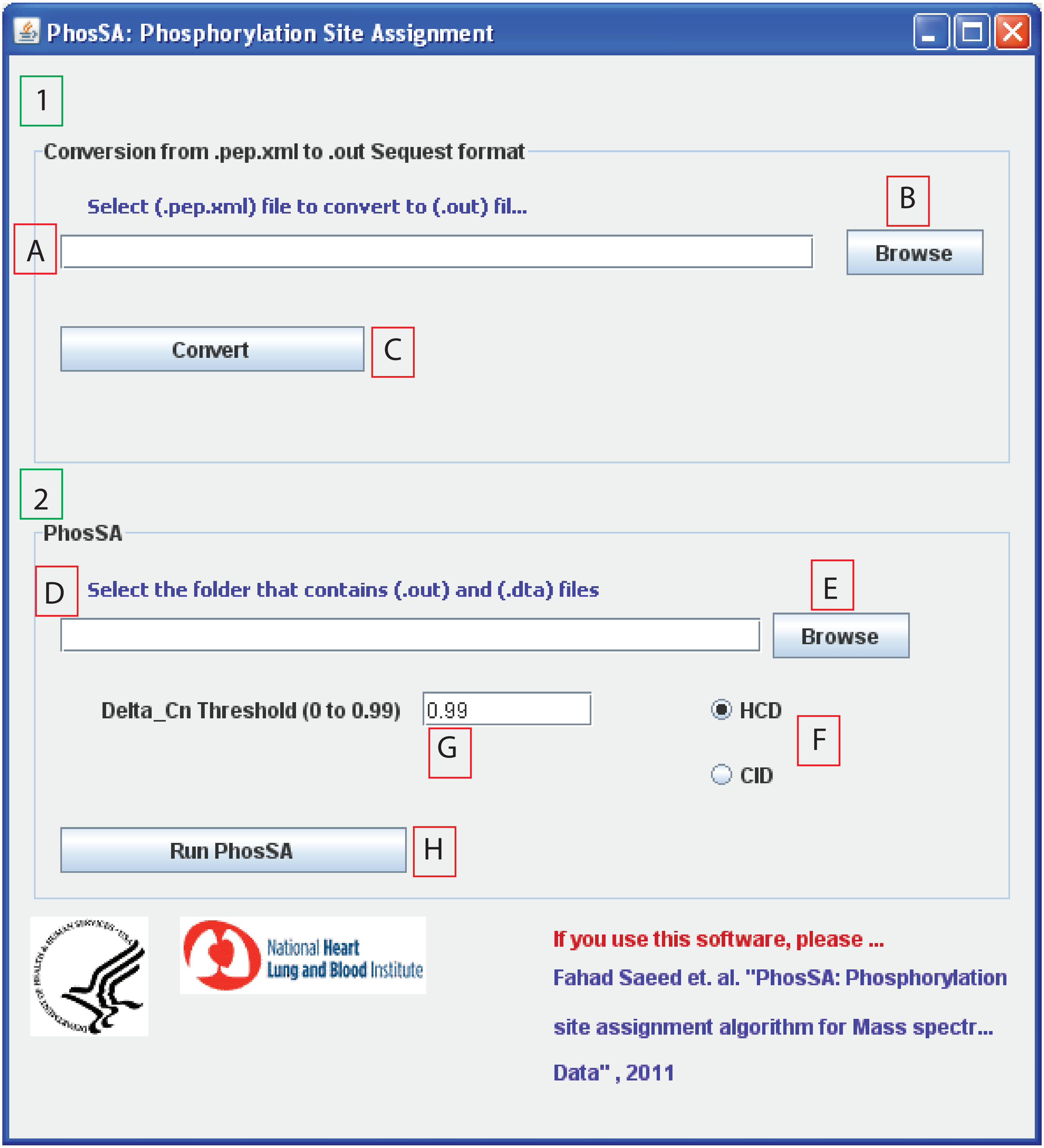
PhosSA: Phosphorylation Site Assignment
1
Conversion from .pep.xml to .out Sequest format
Select (.pep.xml) file to convert to (.out) fil...
A
B
Browse
Convert
C
2
PhosSA
D
Select the folder that contains (.out) and (.dta) files
E
Browse
Delta_Cn Threshold (0 to 0.99)
0.99
G
HCD
CID
F
Run PhosSA
H
National Heart
Lung and Blood Institute
If you use this software, please ...
Fahad Saeed et. al. "PhosSA: Phosphorylation
site assignment algorithm for Mass spectr...
Data" , 2011
