# Supplementary material for: PhosSA: Fast and accurate phosphorylation site assignment algorithm for mass spectrometry data
Source: Proteome Sci. 2013 Nov 7;11(Suppl 1):S14. doi: 10.1186/1477-5956-11-S1-S14 (PMC3909108; doi:10.1186/1477-5956-11-S1-S14)
Supplement: Additional file 15 — Figure S15. The output format in a text file is shown. [file 1477-5956-11-S1-S14-S15.pdf]

A	B	C	D	E
JH_1_HCD.1799.1799.3	K.HPEAPDEES*DHDYQNHI.-	0.92626024	1	Ambiguous
JH_1_HCD.1806.1806.3	R.LGNRKS*VVFTSAR.A	0.92318013	1	Ambiguous
JH_1_HCD.1810.1810.3	K.EELEQQT*DGDCDEEDDDKDGEMPK.S	1	2	Passed
JH_1_HCD.1814.1814.2	K.IGGHGGEYGEEALQR.M	1	1	Passed
JH_1_HCD.1815.1815.2	R.VRPASSAAS*VYAGAGGSGSR.I	0.18312842	2	Ambiguous
JH_1_HCD.1818.1818.3	R.VRPASSAAS*VYAGAGGSGSR.I	0.05426612	2	Ambiguous
JH_1_HCD.1820.1820.3	R.RRQS*VELHS*PQSLPR.G	0.50481409	108	Passed
JH_1_HCD.1821.1821.2	K.EELEQQT*DGDCDEEDDDKDGEMPK.S	1	2	Passed
JH_1_HCD.1845.1845.2	K.LSSPATLNSR.V	1	2	Passed
JH_1_HCD.1867.1867.3	K.AKPS*PAPS*PTISAPDASGPQKR.S	0.04270222	1	Ambiguous
JH_1_HCD.1869.1869.2	R.RRQSVELHS*PQS*LPR.G	0.22501839	1	Ambiguous
JH_1_HCD.1870.1870.3	R.RRQS*VELHSPQSLPR.G	0.58898423	3	Ambiguous
JH_1_HCD.1873.1873.3	R.RRQS*VELHS*PQSLPR.G	0.36523256	108	Passed
JH_1_HCD.1877.1877.4	R.RRQS*VELHSPQSLPR.G	0.8396499	3	Ambiguous
JH_1_HCD.1887.1887.4	R.RRQS*VELHS*PQSLPR.G	0.6618544	108	Passed
JH_1_HCD.1892.1892.2	K.HTGPNS*PDTANDGFVR.L	0.39708935	1	Ambiguous
JH_1_HCD.1905.1905.3	K.GVPM#KARM#IHSLS*GKK.S	0.12197665	2	Ambiguous
JH_1_HCD.1922.1922.2	R.RRQS*VELHS*PQSLPR.G	0.38376987	108	Passed
JH_1_HCD.1925.1925.2	K.YNEVLTQCCTESDK.A	1	1	Passed
JH_1_HCD.1929.1929.3	R.RRQS*VELHS*PQSLPR.G	0.35837882	108	Passed
JH_1_HCD.1936.1936.4	R.RRQS*VELHS*PQSLPR.G	0.69158719	108	Passed
JH_1_HCD.1940.1940.3	R.RRQS*VELHSPQSLPR.G	0.52142149	3	Ambiguous
